# A Lung‐Immune Dual‐Humanized Mouse Using Cryopreserved Tissue Enables Infection and Immune Profiling of Human Common Cold Coronaviruses

**DOI:** 10.1002/advs.202512097

**Published:** 2025-12-07

**Authors:** Chunyu Cheng, Jing Sun, Chun Lu, Qian Yu, Yiyun He, Bingqing Wu, Deshan Ren, Shuai Ding, Jincun Zhao, Yan Li

**Affiliations:** ^1^ MOE Key Laboratory of Model Animal for Disease Study Model Animal Research Center Department of Oncology Nanjing Drum Tower Hospital Affiliated Hospital of Medical School Nanjing University Nanjing 210093 China; ^2^ State Key Laboratory of Respiratory Disease National Clinical Research Centre for Respiratory Disease National Centre for Respiratory Medicine Guangzhou Institute of Respiratory Health the First Affiliated Hospital of Guangzhou Medical University Guangzhou Medical University Guangzhou Guangdong 510182 China; ^3^ School of Basic Medical Sciences Guangzhou Medical University Guangzhou Guangdong 510182 China; ^4^ Department of Rheumatology and Immunology Nanjing Drum Tower Hospital The Affiliated Hospital of Nanjing University Medical School Nanjing 210008 China; ^5^ Guangzhou National Laboratory Guangzhou International Bio Island Guangzhou Guangdong 510005 China; ^6^ Shanghai Institute for Advanced Immunochemical Studies School of Life Science and Technology Shanghai Tech University Shanghai 201210 China; ^7^ Chemistry and Biomedicine Innovation Center (ChemBIC) ChemBioMed Interdisciplinary Research Center Nanjing University Nanjing 210093 China; ^8^ Wuxi Xishan NJU Institute of Applied Biotechnology Wuxi 214000 China

**Keywords:** cryopreserved tissue transplantation; SARS‐CoV2, HKU‐1, human common cold coronaviruses (CCCoVs), humanized mouse model

## Abstract

Human common cold coronaviruses (CCCoVs, e.g., 229E, NL63, OC43, HKU1) hold critical yet underexplored significance in understanding coronavirus evolutionary dynamics and immune cross‐protection, offering insights for predicting emerging pathogens and developing pan‐coronavirus vaccines. However, research is hindered by the lack of animal models due to strict human‐specific tropism and the confounding effects of frequent co‐infections from clinical samples, which obscure virus‐specific pathogenesis. Although lung‐humanized mice have been used in SARS‐CoV‐2 studies, their application to CCCoVs remains unvalidated and relies on logistically challenging fresh human tissues. This study optimizes a transplantation strategy using cryopreserved human fetal lung tissue, achieving enhanced engraftment efficiency. And the refined model supports robust infection by all four major CCCoVs and demonstrates the therapeutic efficacy of Paxlovid against HKU1. Furthermore, comparative analysis reveals phenotypic distinctions in human immune cells between native mouse lungs and human lung implants in lung‐immune dual‐humanized mice. The model also enables validation of virus‐specific T cell responses and assessment of SARS‐CoV‐2 cross‐reactivity post‐HKU1 infection. Overall, this study establishes a scalable platform using cryopreserved tissues for respiratory virus research, overcoming prior limitations in modeling human‐specific tropism and dissecting immune‐pathogen interactions.

## Graphical abstract

Cryopreserved lung‐humanized mice overcome the dependency to fresh tissues and permit head‐to‐head profiling of all four human common cold coronaviruses versus SARS‐CoV‐2 infection; the model validates Paxlovid efficacy against HKU1 and, when coupled with human immune‐system engraftment, enables interrogation of lung‐resident human immunity and HKU1‐induced cross‐reactive T cells to SARS‐CoV‐2.

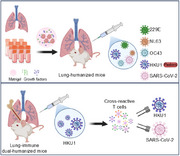

## 1 Introduction

The emergence of novel and previously underestimated viruses highlights their pandemic potential, as exemplified by COVID‐19, which renewed global focus on human coronaviruses (hCoVs). Among the seven known hCoVs, severe acute respiratory syndrome coronavirus (SARS‐CoV), Middle East respiratory syndrome coronavirus (MERS‐CoV), and severe acute respiratory syndrome coronavirus 2 (SARS‐CoV‐2) have been linked to severe lower respiratory tract disease and high mortality rates.^[^ ^1^, ^2^ ^]^ The other four endemic strains (229E, NL63, OC43, and HKU1) cause mostly mild upper respiratory infections and are collectively known as common cold coronaviruses (CCCoVs). 229E and NL63 belong to the *Alphacoronavirus* genus, whereas HKU1, OC43, SARS‐CoV, SARS‐CoV‐2, and MERS‐CoV are members of the *Betacoronavirus* genus. Despite their generally low pathogenicity, CCCoVs are highly prevalent: serological studies indicate that nearly 90% of adults have been exposed to each CCCoV.^[^ ^3^, ^4^ ^]^ These distinct clinical outcomes suggest fundamental differences in viral‐host interactions, particularly in tissue tropism: CCCoVs tend to infect the upper respiratory tract, whereas highly pathogenic hCoVs more often target the lower airways.^[^ ^5^, ^6^ ^]^ Elucidating these divergent tropisms is essential both for understanding how shifts in cellular targeting drive the evolution of virulence and pathology in coronaviruses, and for identifying key host receptors that mediate viral entry.

Interestingly, despite these divergent pathogenic profiles, CCCoVs share approximately 65–69% sequence homology with SARS‐CoV‐2,^[^ ^7^ ^]^ raising the possibility of cross‐reactive immune responses. Characterizing the nature of these responses is critical for elucidating coronavirus immunopathogenesis and guiding the development of next‐generation, broadly protective pan‐coronavirus vaccines. Indeed, multiple studies have identified pre‐existing T cells in unexposed individuals that recognize conserved epitopes shared between CCCoVs and SARS‐CoV‐2.^[^ ^8^, ^9^, ^10^, ^11^, ^12^, ^13^, ^14^ ^]^ However, identifying the specific CCCoV origins of these cross‐reactive T cell responses remains challenging. Individual variability in age, sex, exposure dose, and, most importantly, undocumented infection history complicates efforts to trace the source of such immunity. Moreover, most existing studies rely on peripheral blood mononuclear cells, which provide limited insight into tissue‐resident immune responses. Access to well‐preserved clinical samples and relevant tissue compartments also remains limited, further constraining mechanistic investigations. These challenges highlight the need for well‐controlled experimental animal models capable of supporting productive hCoV infection and recapitulating human‐specific immune responses.

Mice remain the most widely used model in immunological research. However, research on CCCoVs has been hindered by their strict species tropism and substantial differences in immune system. While some transgenic or viral vector‐based mouse models have been constructed to support infection by specific CCCoVs,^[^ ^15^, ^16^ ^]^ they are typically designed for individual viruses and rely on well‐characterized human receptors. Even when infection is established, interspecies immune differences can limit translational relevance.^[^ ^17^, ^18^ ^]^ To overcome these limitations, the lung‐humanized mouse models, created by transplanting human fetal lung tissue into the immunodeficient mice, have emerged as powerful platforms for modeling human respiratory infection.^[^ ^19^, ^20^, ^21^, ^22^, ^23^ ^]^ These models have successfully been used to investigate infections with SARS‐CoV‐2, MERS, and respiratory syncytial virus ^[^ ^23^, ^24^, ^25^, ^26^ ^]^ However, their application to CCCoVs has not been validated. Moreover, the reliance on fresh human fetal lung tissue imposes logistical and supply‐related limitations that hinder widespread use.

In this study, we optimized a transplantation strategy using cryopreserved human fetal lung tissue in immunodeficient mice, overcoming the limitations of relying on fresh human tissue. Using this approach, we established a lung‐humanized mouse model that supports robust infection by both CCCoVs and SARS‐CoV‐2, allowing direct comparison of viral replication kinetics and cell tropism to investigate potential determinants of pathogenicity. To further investigate the origin of cross‐reactive T cell responses, we developed a dual‐humanized model by co‐transplanting autologous human hematopoietic stem cells (HSCs). Following HKU1 exposure, we detected virus‐specific human T cell responses and evaluated their potential cross‐reactivity with SARS‐CoV‐2. Collectively, our work provides a versatile and scalable in vivo system for studying hCoV infection and T cell cross‐reactivity under physiologically relevant conditions.

## 2 Results

### 2.1 Optimized Transplantation Strategy Enhances Engraftment of Cryopreserved Human Fetal Lung Tissue in Recipient Immunodeficient Mice

Cryopreservation offers a stable and secure method for long‐term storage of primary tissue isolates, thereby reducing dependence on freshly obtained human tissues. However, cryopreservation may compromise tissue integrity and engraftment potential, particularly in complex tissues like the human fetal lung. To address this limitation, we referred to published single‐cell transcriptomic studies that highlighted fibroblast growth factor (FGF) and vascular endothelial growth factor A (VEGFA) as critical signaling molecules within the alveolar and airway niches during human fetal lung development.^[^ ^27^ ^]^ Based on these findings, we first tested whether supplementation with these growth factors (FGF/VEGFA) could improve the engraftment of cryopreserved human fetal lung tissue. Specifically, human bFGF (1 µg) and VEGFA (200 ng) were incorporated into 30 µL of Matrigel to promote vascularization and support tissue growth. The concentrations used were slightly higher than those typically employed in tumor transplantation models, in order to enhance penetration into the inner regions of the tissue where diffusion is otherwise limited.^[^ ^28^ ^]^ Given the transient in vivo activity of single‐dose growth factor administration, we focused on evaluating the initial tissue remodeling one week post‐transplantation.

**Figure 1:**
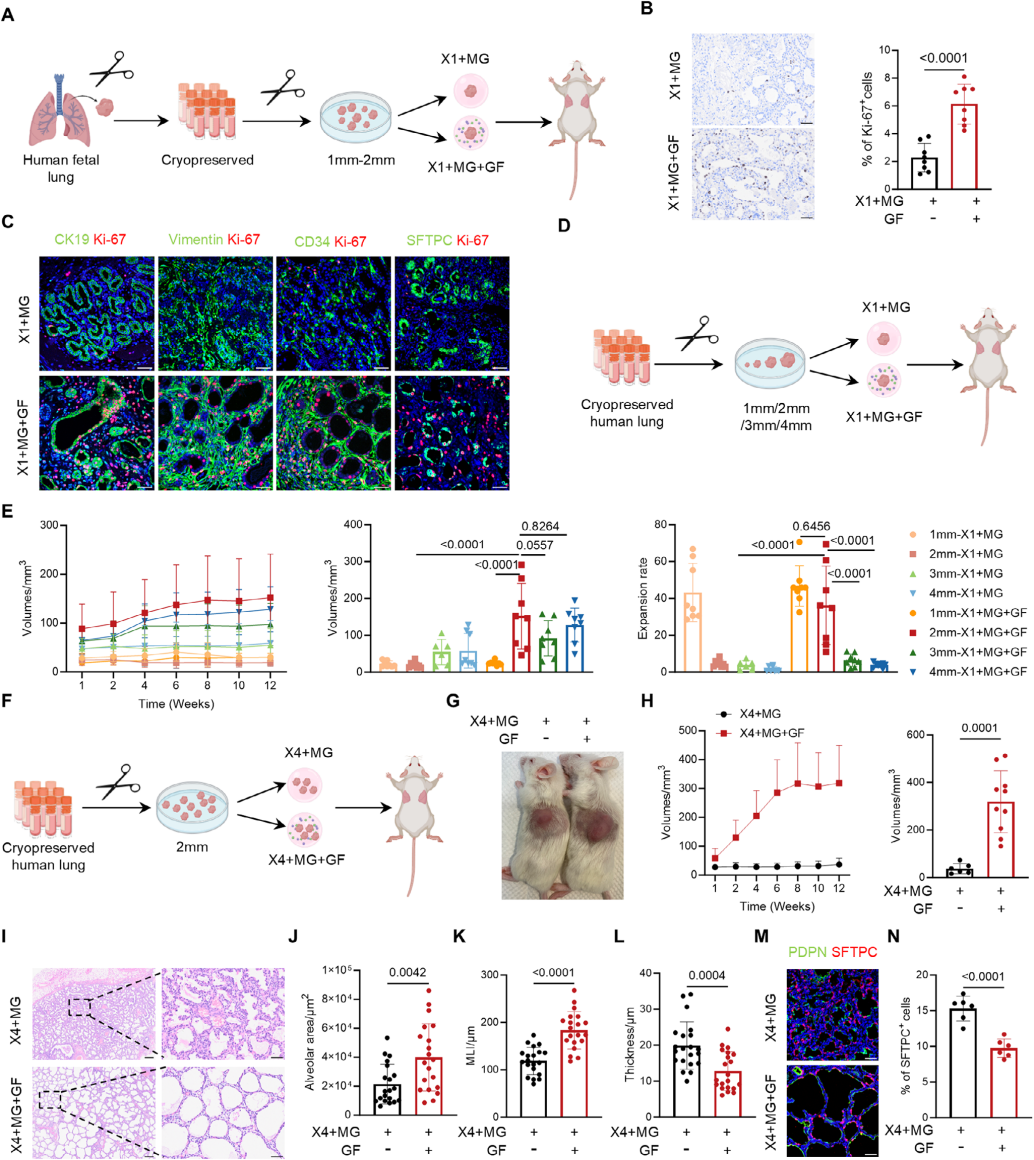
FGF/VEGFA treatment and fragment optimization improve cryopreserved human lung implants engraftment and alveolar remodeling. A) Schematic diagram illustrating the cryopreservation process of human fetal lung tissue, preparation of tissue fragments, treatment strategies, and subcutaneous implantation into immunodeficient mice. B) Immunohistochemical staining for Ki‐67 to evaluate proliferative activity one week after transplantation. Scale bars: 50 µm. n = 8 biological replicates. C) Co‐immunofluorescence staining of Ki‐67 (red) with CK19 (epithelial cells, green), Vimentin (mesenchymal cells, green), CD34 (endothelial cells, green), SFTPC (alveolar type 2 cells, AT2, green). Scale bars: 50 µm. D) Schematic illustrating transplantation of fragments with varying diameters to evaluate the effects of growth factor supplementation and fragment size. E) Growth kinetics, endpoint volumes, and fold expansion (relative to the initial volume) of human lung implants with different fragment diameters and treatments over 12 weeks. Group sizes: 3 mm × 1 + MG, n = 6; all other groups, n = 8. F) Schematic diagram showing transplantation of four 2 mm fragments to evaluate the effect of growth factor treatment. G) Representative images of human lung implants 12 weeks post‐transplantation with or without growth factor treatment. H) Growth curves and endpoint volumes of implants. ×4 + MG, n = 6; ×4 + MG + GF, n = 10. I) H&E staining comparing lung implants with or without growth factor treatment at 12 weeks post‐transplantation. Scale bars: 200 µm (left) and 50 µm (right). J–L) Quantification of (J) alveolar area, (K) mean linear intercept (MLI), and (L) alveolar wall thickness. n = 4 biological replicates, 20 technical replicates. M) Immunofluorescence staining of alveolar type 1 cells (AT1, PDPN, green) and AT2 (SFTPC, red) after 12 weeks of transplantation. Scale bars: 50 µm. N) Quantification of the percentage of SFTPC⁺ AT2. n = 6 biological replicates. Data presented as means ± SD. Statistical analyses were performed using a nonpaired two‐tailed Student's *t*‐test (B,H,J–L,N) and one‐way ANOVA followed by Tukey's multiple comparisons tests for (E). ×1: One cryopreserved human fetal lung tissue fragment. MG: Matrigel. GF: Growth factors. ×4: Four cryopreserved human fetal lung tissue fragments.

Cryopreserved human fetal lung tissues were dissected into small fragments and transplanted subcutaneously into the dorsal flanks of severe immunodeficient mice NCG (NOD‐*Prkdc* ^em26Cd52^ *Il2rg* ^em26Cd22^/Gpt), with one fragment (approximately 1–2 mm in diameter) per flank, as previously described.^[^ ^23^, ^26^ ^]^ Each fragment was embedded in a Matrigel pellet, either with or without growth factors supplementation (**Figure** **1**A). Hematoxylin and eosin (H&E) staining showed no significant morphological differences between groups (Figure S1A, Supporting Information), which was further verified by quantitative histological analyses, including alveolar area, mean linear intercept (MLI), and median alveolar wall thickness (Figure S1B–D, Supporting Information). However, immunostaining for Ki‐67 revealed a significant increase in proliferative activity in the growth factor‐treated group (Figure 1B). Co‐staining with cell lineage markers further demonstrated that this proliferative boost was broadly distributed across epithelial, stromal, endothelial, and alveolar type 2 (AT2) populations (Figure 1C). Consistent with these observations, real‐time quantitative PCR (qPCR) analysis further confirmed the upregulation of genes involved in both cell proliferation and alveolar differentiation (Figure S1E, Supporting Information), highlighting the dual role of FGF and VEGFA in enhancing early‐stage tissue expansion and initiating alveolar maturation in human lung implants.

In addition to signaling support, prior studies have indicated that fragment diameter directly correlates with engraftment efficiency.^[^ ^29^ ^]^ However, existing studies revealed significant variability in the diameters of fresh fetal lung tissue fragments used in transplantation, typically ranging from 1 to 5 mm, with no standardized approach, particularly for cryopreserved tissue.^[^ ^19^, ^22^, ^23^, ^25^, ^30^ ^]^ To systematically investigate the influence of fragment size and growth factor supplementation, we designed an experimental framework using tissue fragments ranging from 1 to 4 mm in diameter, with or without FGF/VEGFA supplementation (Figure 1D). Implant growth dynamics were monitored longitudinally at multiple time points (weeks 1, 2, 4, 6, 8, 10, and 12; Figure 1E). Final implant volumes and fold expansion relative to the initial volume were calculated. Across all tested fragment sizes, growth factor supplementation significantly enhanced tissue expansion, except in the 1 mm group, which showed limited growth regardless of treatment. Notably, 2 mm fragments demonstrated the greatest expansion, indicating that this size in combination with growth factors represents the optimal condition for regenerating cryopreserved lung tissue.

To further enhance engraftment, we next increased the number of transplanted fragments per site. Under the optimized condition (2 mm fragments + growth factors), four fragments were placed on each dorsal flank (Figure 1F). This approach led to rapid fusion into larger masses (Figure 1G) and a significant increase in total volume, which stabilized by week 8 (Figure 1H), as compared to no growth factor supplementation. Histological analysis revealed growth factor‐treated implants displayed enhanced structural maturation (Figure 1I), characterized by expanded alveolar area, improved MLI and thinning of the alveolar septa (Figure 1J–L). Furthermore, these implants also showed a significant reduction in SFTPC⁺ AT2 cells (Figure 1M,N), suggesting accelerated differentiation toward alveolar type 1 (AT1) cells and enhanced alveolar maturation.

In summary, our results demonstrate that growth factor supplementation, fragment size, and number optimization are key strategies to improve the engraftment of cryopreserved human fetal lung tissue. FGF and VEGFA not only enhance early proliferation across multiple cell types but also facilitate alveolar differentiation and maturation, leading to improved implant expansion and structural development.

### 2.2 Structural and Cellular Maturation of Cryopreserved Human Lung Implants Following Subcutaneous Transplantation

**Figure 2:**
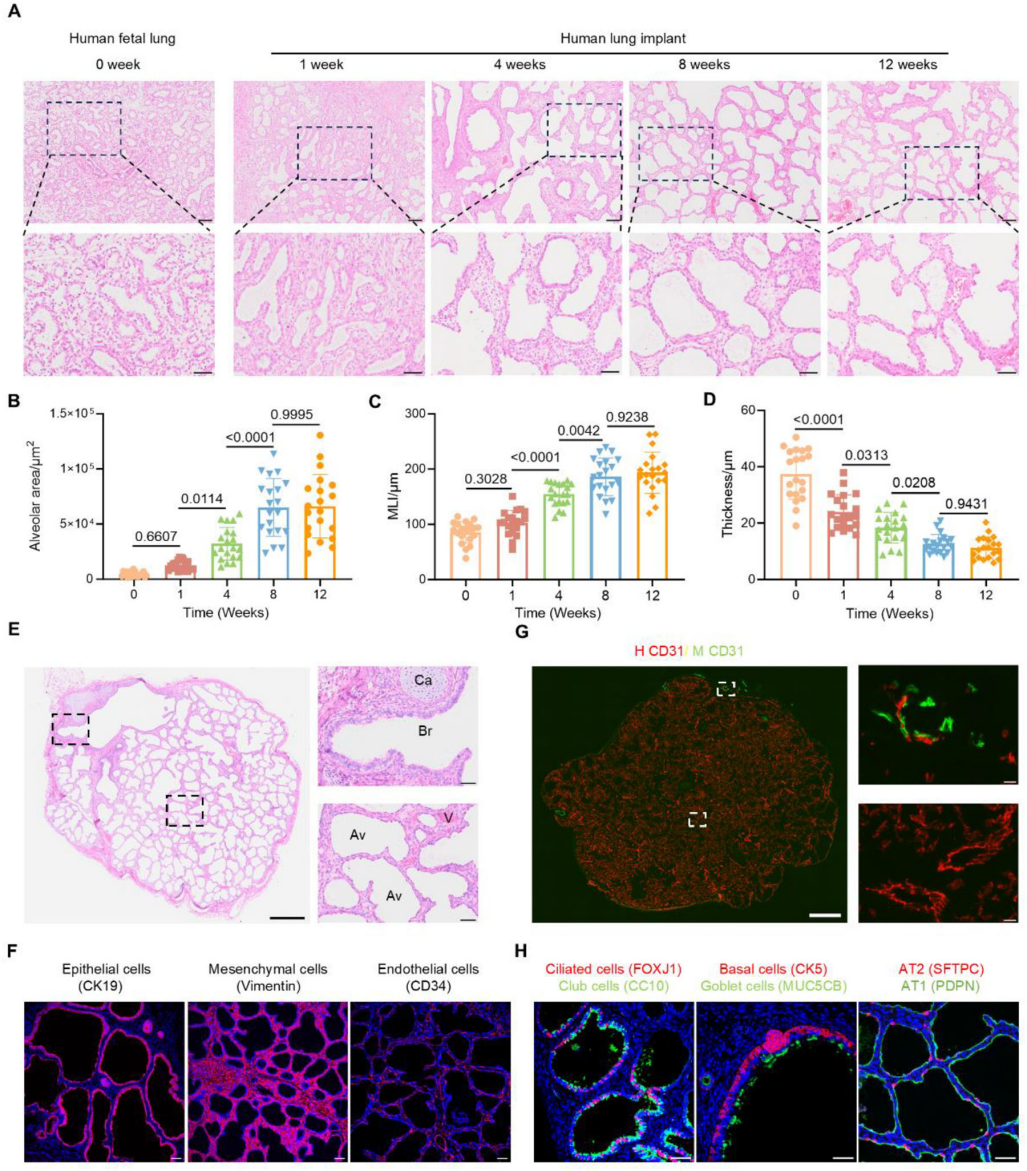
Progressive structural maturation and multilineage differentiation in cryopreserved human lung implants. A) H&E staining showing changes in the alveolar regions of human fetal lung tissue before transplantation and at 1, 4, 8, and 12 weeks after transplantation. Scale bars: 100 µm (top) and 50 µm (down). B–D) Quantification of (B) alveolar area, (C) MLI, and (D) alveolar wall thickness. n = 4 biological replicates, 20 technical replicates. E) Representative H&E staining of human lung implants displaying bronchioles (Br), alveolar structures (Av), cartilage (Ca), and blood vessels (V). Scale bars: 200 µm (left) and 50 µm (right). F) Immunofluorescence analysis of cellular composition in subcutaneous implants, identifying human‐derived epithelial cells (CK19), mesenchymal cells (Vimentin), and endothelial cells (CD34). Scale bars: 50 µm. G) Immunofluorescence staining for human (H CD31) and mouse (M CD31) endothelial cells in human lung implants. Scale bars: 200 µm (left) and 50 µm (right). H) Immunofluorescence staining of airway and alveolar cell subsets, including ciliated cells (FOXJ1), club cells (CC10), basal cell (CK5), goblet cells (MUC5B), AT1 (PDPN), and AT2 (SFTPC). Scale bars: 50 µm. Data presented as means ± SD. Statistical analyses were performed using one‐way ANOVA followed by Tukey's multiple comparisons tests.

Next, we evaluated the structural organization and cellular composition of implants derived from cryopreserved human fetal lung tissue. Prior to transplantation, the fetal lung tissue exhibited hallmark features of the pseudoglandular to early canalicular stages, including small, irregularly shaped airspaces lined by cuboidal epithelial cells and separated by thick, cellular interstitial septa.^[^ ^31^ ^]^ 12 weeks post‐transplantation, the implants underwent progressive structural remodeling, from early luminal expansion and epithelial thinning to the formation of spherical sac‐like airspaces, and eventually acquiring late saccular or early alveolar stage features, including flattened epithelium and thinned interalveolar septa (**Figure** **2**A). Quantitative measurements confirmed these structural changes. Alveolar area and MLI significantly increased from week 1 to 8 before plateauing, while alveolar wall thickness progressively declined and stabilized by week 8 (Figure 2B–D), consistent with ongoing alveolar expansion and septal thinning. These results suggest that cryopreserved lung implants undergo dynamic remodeling toward mature alveolar‐like structures over time. Importantly, all implants retained intact histological integrity with no evidence of degeneration or necrosis.

Further histological analysis revealed well‐organized pulmonary structures, including airways with or without cartilage, dense vasculature, and alveoli with thin interalveolar septa, indicative of substantial developmental maturation (Figure 2E). Immunofluorescence analysis confirmed the human origin of major cell populations, including epithelial, endothelial, and mesenchymal cells within the implants (Figure 2F). Mapping of vascular structures using species‐specific CD31 antibodies revealed that the majority of blood vessels were donor‐derived, although human‐mouse chimeric vessels were occasionally detected at the periphery of implant (Figure 2G). Further profiling of epithelial subtypes revealed a diverse array of mature subsets, including AT1, AT2, ciliated, goblet, club, and basal cells (Figure 2H), effectively recapitulating the cellular heterogeneity of native human lung tissue, consistent with previous reports using freshly isolated human fetal lung tissue for transplantation.^[^ ^23^ ^]^ Together, these findings demonstrate the progressive structural and cellular maturation of cryopreserved human fetal lung tissue following subcutaneous transplantation, supporting its utility as a physiologically relevant platform for human lung research.

To further explore the potential applications of human lung tissue, we adopted a serial transplantation strategy inspired by liver humanized mouse models.^[^ ^32^ ^]^ Human lung tissue harvested from established lung‐humanized mice was transplanted into recipient NCG mice with either a single fragment per flank or, in our optimized protocol, four 2 mm fragments per flank combined with growth factor supplementation (Figure S2A, Supporting Information). After 8 weeks, tissue growth remained minimal, and endpoint volumes showed no significant benefit from growth factor treatment (Figure S2B, Supporting Information). These data reflect the maturation of lung tissue: after initial engraftment, fetal lung tissue matures into adult‐like structures, which are known to possess limited regenerative capacity. Although progenitor populations such as basal, club, and AT2 cells can contribute to regeneration under injury conditions, our subcutaneous implantation model may not provide the requisite injury signals or microenvironmental cues to initiate these repair programs. These findings further indicate that the transplanted human lung implants progressively acquire features of mature human lung tissue.

### 2.3 CCCoV and SARS‐CoV‐2 Displayed Distinct Infection Patterns in Lung‐Humanized Mice

**Figure 3:**
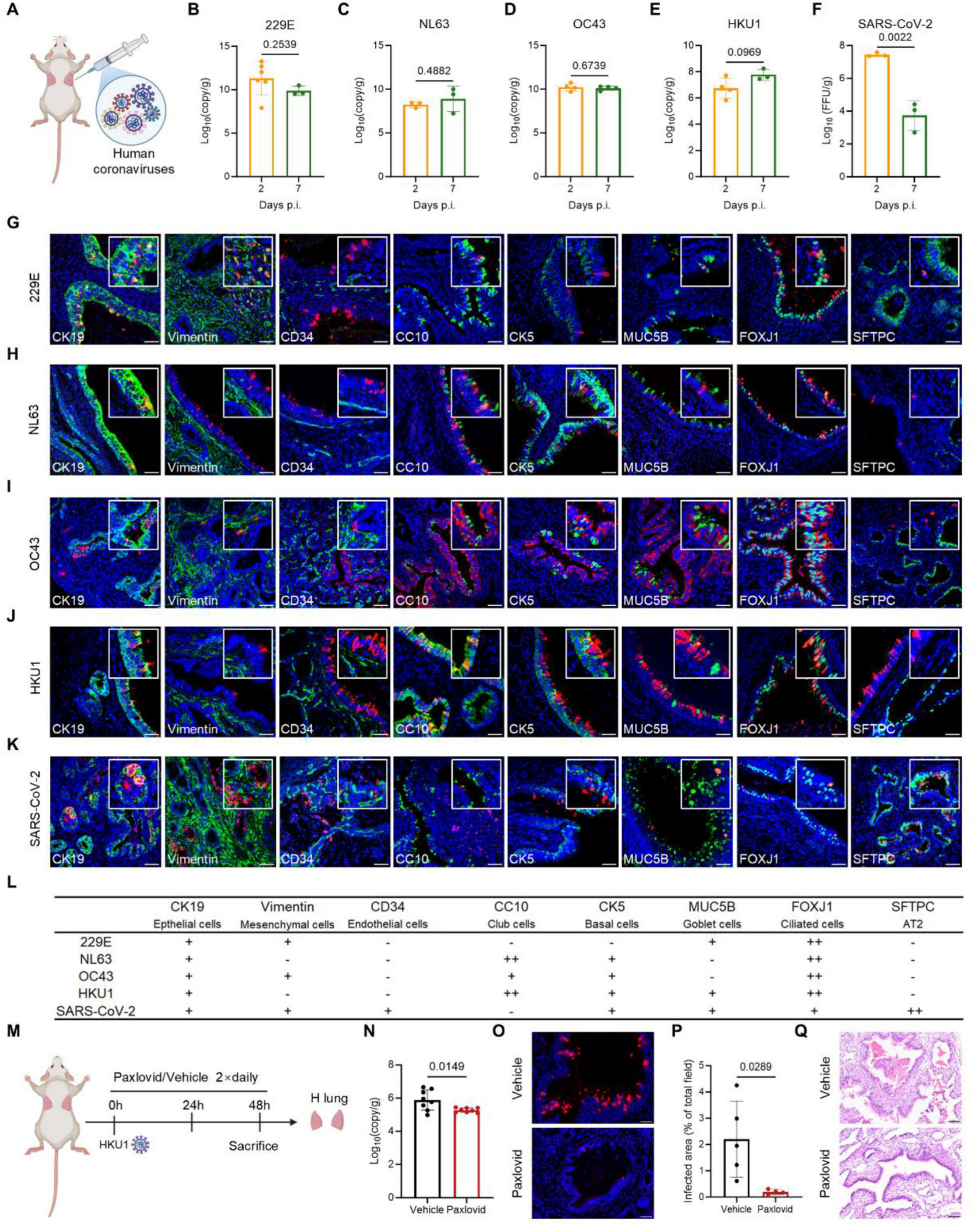
Differential infection kinetics and cellular tropism of CCCoVs and SARS‐CoV‐2 in human lung implants. A) Schematic diagram illustrating of human lung implant infection by multiple human coronaviruses. B–F) Viral titers in infected human lung implants: B) 229E at 2 d (n = 6) and 7 d (n = 3). C) NL63 at 2 d (n = 3) and 7 d (n = 3). D) OC43 at 2 d (n = 4) and 7 d (n = 4). E) HKU1 at 2 d (n = 4) and 7 d (n = 3). F) SARS‐CoV‐2 at 2 d (n = 3) and 7 d (n = 3). G–K) Representative immunofluorescence co‐staining showing virus‐infected cells (red) and markers of specific human cell types (green): CK19 (epithelial cells), vimentin (mesenchymal cells), CD34 (endothelial cells), CC10 (club cells), CK5 (basal cells), MUC5B (goblet cells) and FOXJ1 (ciliated cells), SFTPC (AT2). Scale bars: 50 µm. L) Summary of the susceptible cell subtypes for 229E, NL63, OC43, HKU1, and SARS‐CoV‐2 shown in panels (G–K). M) Schematic of the experimental design. Lung‐humanized mice were orally administered Paxlovid or vehicle starting at the time of HKU1 exposure (0 h) followed by dosing every 12 hours. Human lung implants were harvested 48 h after infection. N) HKU1 titers in the human lung implants administered Paxlovid (n = 8) or vehicle (n = 8) 48 h after exposure to virus. O) Representative immunofluorescence staining of viral nucleoprotein in human lung implants from Paxlovid‐ and vehicle‐treated mice. Scale bars: 50 µm. P) Quantification of HKU1 infected area as a percentage of the total field in Paxlovid‐treated (n = 4) and vehicle‐treated (n = 5) groups. Q) Representative histopathological features of HKU1 infected human lung implants following Paxlovid or vehicle treatment. Scale bars: 50 µm. Data presented as means ± SD. Statistical analyses were performed using a nonpaired two‐tailed Student's *t*‐test.

To evaluate viral replication and cellular tropism, we inoculated subcutaneous human lung implants with four major CCCoVs (229E, NL63, OC43, and HKU1) as well as SARS‐CoV‐2 (**Figure** **3**A). Four CCCoVs maintained consistent viral titers from day 2 to day 7 post‐infection, suggesting limited viral clearance within this period (Figure 3B–E). In contrast, SARS‐CoV‐2 exhibited a distinct viral kinetic pattern, with peak titers at day 2, followed by a sharp decline by day 7 (Figure 3F), consistent with previous reports.^[^ ^26^ ^]^ Immunohistochemistry staining confirmed successful infection across all tested hCoVs, while revealing marked differences in tissue tropism. The four CCCoVs predominantly localized to airway regions, whereas SARS‐CoV‐2 infection was mainly restricted to alveolar compartments (Figure S3A, Supporting Information), highlighting fundamental differences in the pathogenesis of low‐versus high‐pathogenicity hCoVs.

Cellular tropism analysis further demonstrated distinct viral preferences. Immunofluorescence co‐staining revealed that all four CCCoVs primarily infected epithelial cells (Figure 3G–J). Among them, 229E and OC43 also targeted mesenchymal cells (Figure 3G,I), while none of the CCCoVs infected endothelial cells. Notably, 229E exhibited broad tropism, targeting mesenchymal, goblet cells, and ciliated cells (Figure 3G). NL63, OC43, and HKU1 demonstrated consistent tropism for basal and club cells, with NL63 and HKU1 showing particularly strong association with club cells (Figure 3H–J). All four CCCoVs displayed strong tropism for ciliated cells, as evidenced by robust FOXJ1 expression (Figure 3G–J). Collectively, these findings suggest although ciliated cells serve as a common target, virus‐specific preferences for other epithelial subtypes drive differential cellular susceptibility. In comparison, SARS‐CoV‐2 exhibited a markedly broader and more diverse cellular tropism (Figure 3K). Similar to 229E and OC43, it infected both epithelial and mesenchymal cells. However, it uniquely targeted endothelial cells and showed strong tropism for AT2 cells (Figure 3K,L). This expanded tropism across both airway and alveolar compartments likely contributes to its higher pathogenic potential.

Histopathological examination at day 7 post‐infection revealed varying degrees of tissue injury. All four CCCoVs elicited significant immune infiltration (predominantly neutrophils) and epithelial barrier disruption, evidenced by detaching or sloughing off from the airway epithelium (Figure S3B, Supporting Information). While SARS‐CoV‐2 induced similar histopathological features, but with markedly amplified severity. Notable pathological findings included alveolar hemorrhage, extensive fibrin thrombi occluding blood vessels, and the accumulation of necrotic debris within airspaces (Figure S3B, Supporting Information), which closely recapitulate the severe lung pathology in critical COVID‐19.^[^ ^33^, ^34^ ^]^ Together, these results demonstrate that CCCoVs and SARS‐CoV‐2 exhibit distinct viral dynamics, cellular tropism, and tissue pathology in human lung implants, reflecting their divergent pathogenic profiles in human disease.

### 2.4 Paxlovid Protects Against HKU1 Infection In Vivo

Although CCCoVs such as HKU1 are generally associated with mild respiratory illness, our histopathological analysis revealed that these CCCoVs can still induce mild to moderate airway epithelial injury. To date, no antiviral treatments are approved for CCCoV infections, highlighting a significant unmet clinical need. Given that the 3CL protease targeted by nirmatrelvir (a key component of Paxlovid) is highly conserved across hCoVs,^[^ ^35^, ^36^ ^]^ we next investigated the therapeutic potential of Paxlovid against HKU1 infection. Lung‐humanized mice were orally administered Paxlovid or vehicle beginning at the time of viral exposure (0 h), with dosing every 12 hours thereafter (Figure 3M). Human lung implants were harvested 48 h after infection for analysis. Viral titer measurements showed a marked reduction in the Paxlovid‐treated group (Figure 3N). Immunofluorescence analysis further confirmed that Paxlovid treatment significantly suppressed HKU1 viral replication within human lung implants (Figure 3O,P). In contrast, vehicle‐treated group exhibited abundant viral nucleoprotein‐positive cells and widespread cellular debris within the airway lumen (Figure 3O–Q), indicating active viral replication and epithelial injury. These results demonstrate that lung‐humanized mice can robustly support HKU1 infection and provide a tractable in vivo platform for evaluating antiviral therapies targeting CCCoVs.

### 2.5 Human Lung Stromal Niches Promotes Human Immune Cell Engraftment and Maturation

**Figure 4:**
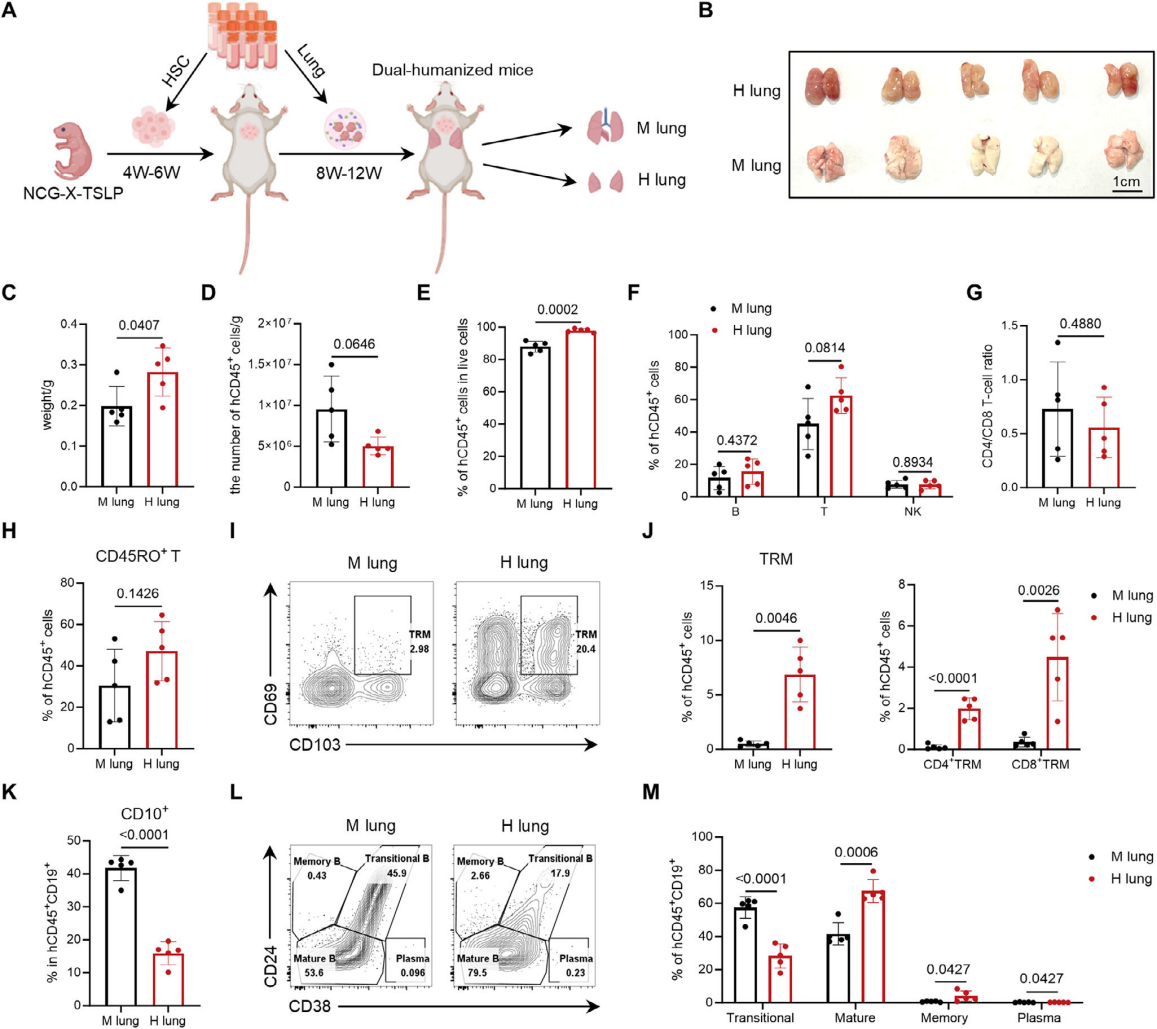
Comparative analysis reveals distinct human immune composition between mouse lungs and human lung implants. A) Schematic diagram illustrating the construction of lung‐immune system dual‐humanized mice using cryopreserved human fetal lung tissue. B) Representative images of lung tissues. M lung: mouse lung tissue. H lung: human lung implant. C) Weights of mouse lung tissue (M lung) and human lung implants (H lung), along with the D) absolute numbers of human immune cells per gram of tissue. E) Percentage of human immune cells (hCD45^+^) in both tissues. F) Percentage of human immune cell subsets, including T cells (hCD3^+^), B cells (hCD19^+^), and NK cells (hCD56^+^). G) T Ratio of human CD4^+^ to CD8^+^ T cells. H) Percentage of human memory T cells (hCD45RO^+^) in both tissues. I) Representative flow cytometry plots showing the percentage of tissue‐resident memory T cells (TRM, hCD45RO^+^ hCD69^+^ hCD103^+^). J) Percentage of human TRM, CD4^+^ TRM, and CD8^+^ TRM in both tissues. K) Percentage of hCD10^+^ B cells in both tissues. L) Representative flow cytometry analysis and the M) quantification of human B cell subsets, including transitional B cells (hCD24^high^ hCD38^high^), mature B cells (hCD24^int^ hCD38^int^), memory B cells (hCD24^high^ hCD38^−^) and plasmablasts (hCD24^−^ hCD38^high^). Data presented as means ± SD. Statistical analyses were performed using unpaired two‐tailed Student's *t*‐test. n = 5 biological replicates.

We then constructed a dual‐humanized mouse model by co‐transplanting autologous human HSCs and cryopreserved human fetal lung implants into immunodeficient mice (**Figure** **4**A), following our optimized protocol above. We used NCG‐X‐TSLP (NOD‐*Prkdc* ^em26Cd52^ *Il2rg* ^em26Cd22^ *kit^em1Cin(V831M^Gpt‐Tg(mTSLP)918/*Gpt) mice with induced expression of thymic stromal lymphopoietin (TSLP) as recipients. Our previous study have shown that TSLP expression promotes the development of secondary lymphoid tissues, which are essential for supporting human immune cell maturation and antigen‐specific responses in vivo.^[^ ^37^ ^]^ Given the known role of human stromal components in regulating immune cell development and function,^[^ ^38^ ^]^ we sought to compare the phenotypes of human immune cells between the murine‐derived (host) and human‐derived (implant) lung compartments within these dual‐humanized mice.

Morphologically, human lung implants were comparable in size to the murine lungs (Figure 4B). Quantitative assessment showed that the human lung implants displayed slightly higher tissue weight (Figure 4C), while the density of human immune cells remained similar between the two compartments (Figure 4D). Flow cytometry analysis revealed a significantly higher proportion of total human immune cell infiltration in the human lung implants compared to murine lungs (Figure 4E). Lymphoid cell subsets‐including T cells, B cells, and natural killer (NK) cells‐were similarly distributed between compartments (Figure 4F; Figure S4A, Supporting Information). Immunohistochemistry further confirmed abundant human immune cell presence within the human lung implants (Figure S4B, Supporting Information). Detailed immunophenotyping showed comparable CD4/CD8 T cell ratios (Figure 4G) and similar distributions of T cell subsets, including conventional αβ T cells, γδ T cells, mucosal‐associated invariant T cells (MAIT) cells, and natural killer T (NKT) cells, between the two compartments (Figure S4C, Supporting Information). The frequency of regulatory T cells (Tregs) was also comparable (Figure S4D,E, Supporting Information). Functional analysis via Phorbol 12‐myristate‐13‐acetate (PMA)/ionomycin stimulation demonstrated equivalent cytokine and chemokine production profiles by human T cells from both lung compartments (Figure S4F, Supporting Information).

CD45RO^+^ memory T cells were present at comparable frequencies in both compartments (Figure 4H). However, tissue‐resident memory T (TRM) cells, defined by co‐expression of CD45RO, CD69, and CD103, as reported previously,^[^ ^39^, ^40^ ^]^ were significantly enriched in the human lung implants across both CD4⁺ and CD8⁺ subsets (Figure 4I,J). In the B cell compartment, human lung implants showed a more mature phenotype, as compared to mouse lung. B cells from the implants expressed lower levels of CD10 (Figure 4K), a marker of early B cell development,^[^ ^41^ ^]^ and exhibited a reduced proportion of transitional B cells alongside an increased frequency of mature B cells (Figure 4L,M). This phenotype was further supported by an elevated proportion of CD27^−^IgD⁺ B cells, consistent with a mature state (Figure S5A,B, Supporting Information). Collectively, these results demonstrate that the human stromal microenvironment within the lung implants not only supports the engraftment of human immune cells but also promotes their phenotypic maturation and differentiation.

### 2.6 Human Macrophages and TRMs are Robustly Reconstituted in Human Lung Implants of Dual‐Humanized Mice

**Figure 5:**
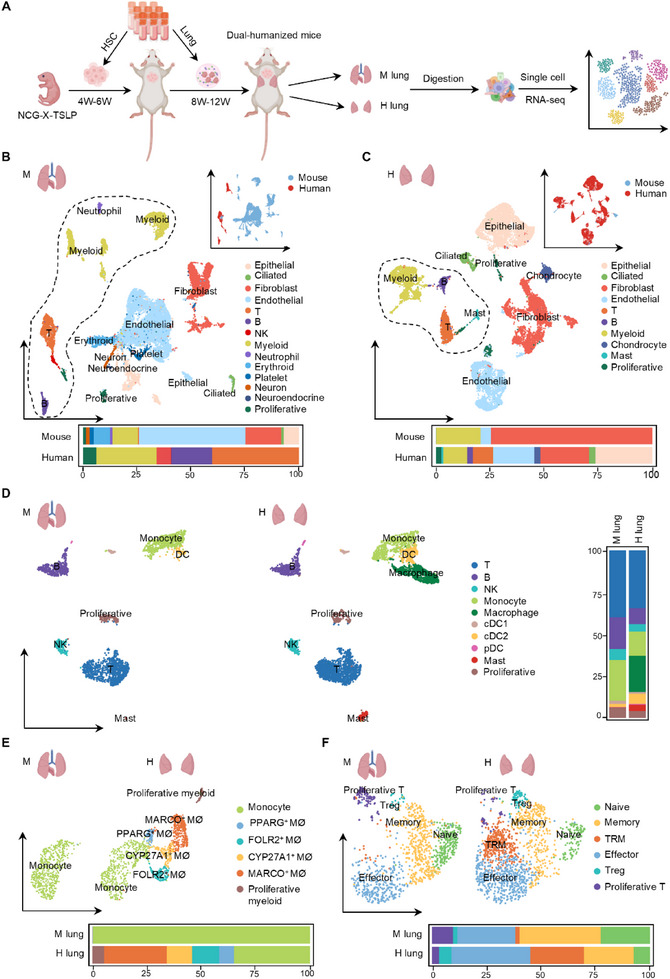
Human lung implants support selective enrichment of macrophages and TRMs. A) Schematic overview of the scRNA‐seq workflow. B,C) UMAP depicting the distribution of stromal and immune cells in (B) mouse lungs and (C) human lung implants. Frequencies of cell subsets within the human and mouse cell compartments are shown below the respective UMAP plots. D) UMAP plots and subpopulation frequencies of human immune cells identified in mouse lungs and human lung implants. Frequencies of cell subsets are shown near the respective UMAP plots (cDC, conventional dendritic cells; pDC, plasmacytoid dendritic cells). E,F) UMAP plots and subclustering analyses of human (E) myeloid cells and (F) T cells in mouse lungs and lung implants. Frequencies of cell subsets are shown below the respective UMAP plots (MØ, marophages).

To systematically compare human and murine lung compartments, we performed single‐cell RNA sequencing (scRNA‐seq) on lung‐immune system dual‐humanized mice (**Figure** **5**A). Transcriptomic analysis revealed comparable cellular complexity across human lung implants and murine lungs, with core cellular lineages including epithelial, endothelial, fibroblast, and immune subsets (Figure 5B,C). Of note, certain cell types exhibited species‐specific distribution: neuron‐associated cells and platelets were uniquely detected in murine lungs, whereas chondrocytes were exclusively identified in human lung implants. Within the epithelial compartment, both tissues contained AT1 and AT2, as well as club and ciliated cells (Figure S6A,B, Supporting Information). Notably, basal cells were markedly enriched in human lung implants, consistent with known species‐specific differences: as basal cells are limited to the trachea in mice but broadly distributed across the human airway epithelium, including distal bronchioles.^[^ ^42^, ^43^ ^]^

We next focused on the distribution of human immune cells across compartments. A striking observation was the robust enrichment of human macrophages in the human lung implants but not the murine lungs (Figure 5D). Subclustering of these macrophages identified four transcriptionally distinct subsets‐PPARG^+^, FOLR2^+^, CYP27A1^+^, and MARCO⁺‐each representing potentially specialized functions within the human lung microenvironment (Figure 5E; Figure S6C, Supporting Information). In parallel, we examined the distribution and phenotype of TRMs. Consistent with flow cytometry data, TRMs were significantly enriched in the human lung implants and expressed canonical markers such as CD69, ITGAE (CD103), and ITGA1 (CD49a) (Figure 5F; Figure S6D, Supporting Information), supporting their establishment and maintenance in the human‐derived lung environment. Additionally, consistent with flow cytometry results, the human lung implants exhibited a higher proportion of mature B cells compared to mouse lungs (Figure S6E,F, Supporting Information). These observations collectively underscore the robust establishment of human innate and adaptive immunity within the implanted lung tissue, particularly the enrichment of human macrophages and TRMs that may contribute to local immune surveillance and tissue homeostasis.

### 2.7 Alveolar Fibroblasts and FOLR2^+^ Macrophages Cooperatively Sustain TRM Maintenance in Human Lung Implants

**Figure 6:**
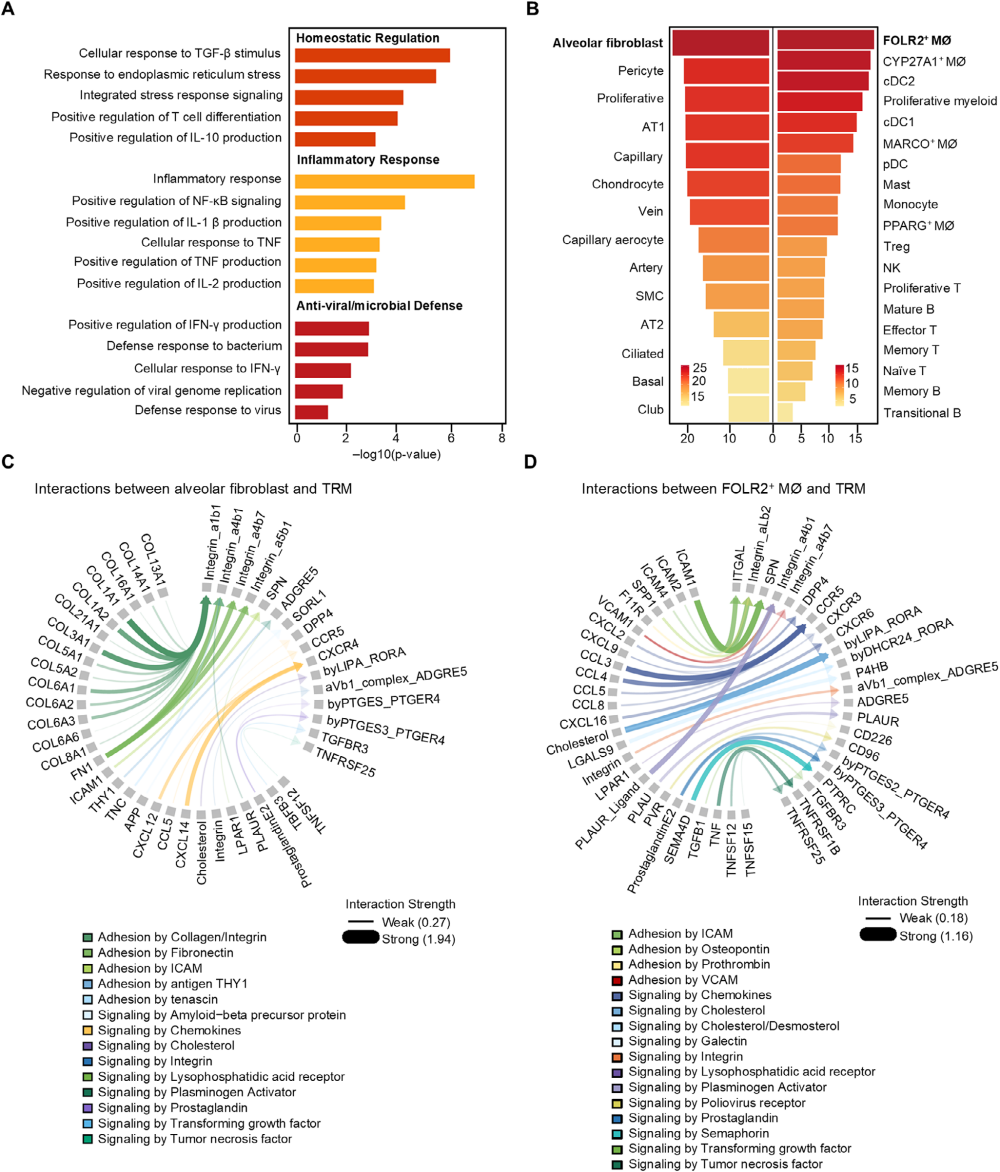
Stromal and immune cell interactions sustain TRM in human lung implants. A) Gene Ontology term enrichment analysis showing signaling pathways of TRM in human lung implants. B) Overall signaling strength from stromal and immune populations targeting TRM. C,D) Ligand‐receptor interactions from (C) alveolar fibroblasts and (D) FOLR2⁺ macrophages to TRM.

To better understand the functional programs of these TRMs and the niche‐derived signals that support their maintenance, we next performed transcriptomic and cell‐cell interaction analyses focused on the TRM compartment. Functional enrichment analysis of TRM‐associated genes identified three core modules‐antiviral defense, cytokine‐mediated inflammation, and homeostatic regulation‐highlighting their dual roles in immune activation and long‐term tissue residency (**Figure** **6**A). To explore the niche‐derived signals that regulate TRM maintenance, we conducted a cell‐cell communication analysis using TRMs as the receiving population. Among the interacting cell types, FOLR2⁺ macrophages represented the strongest immune‐derived regulators, while alveolar fibroblasts were the most dominant stromal contributors (Figure 6B). Alveolar fibroblasts primarily contributed through structural and adhesive cues, particularly collagen‐integrin and fibronectin‐integrin interactions, whereas FOLR2⁺ macrophages provided diverse immunomodulatory signals, including chemokines, TNF superfamily members, and cholesterol‐associated ligands (Figure 6C,D). Importantly, both cell types converged on integrin‐mediated adhesion pathways, which represented the strongest and most conserved signaling axis targeting TRMs. These integrin interactions likely play a central role in anchoring TRMs within the lung niche and promoting their long‐term maintenance. Together, these findings highlight the coordinated roles of stromal and immune cells in shaping the TRM in the human lung microenvironment.

### 2.8 Lung‐Immune Dual‐Humanized Mice Support Functional Dendritic Cell (DC)‐T Cell Interactions and *De Novo* Virus‐Specific T Cell Responses

**Figure 7:**
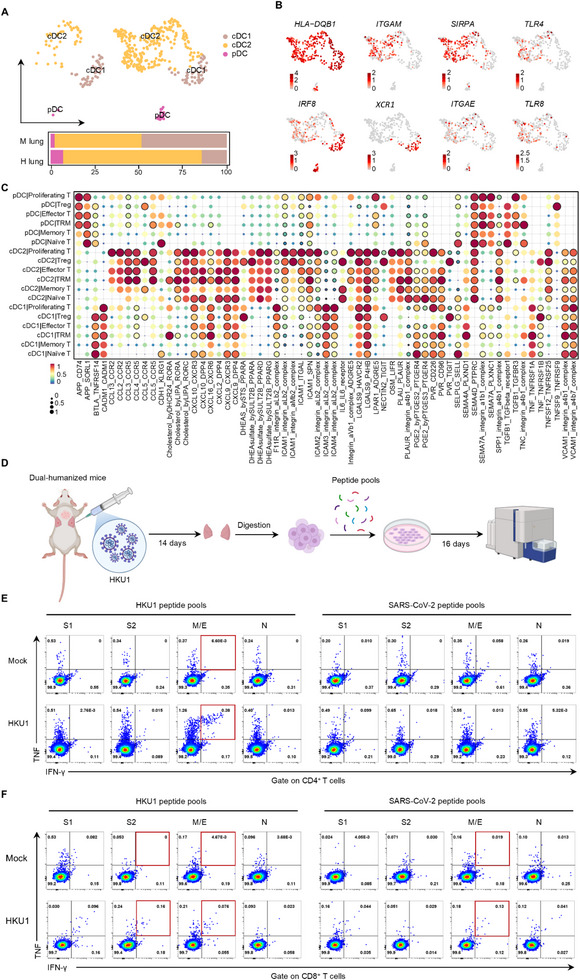
DC‐T cell crosstalk and de novo HKU1‐specific T cell responses in lung‐immune system dual‐humanized mice. A) UMAP visualization of DC subsets identified in murine lung (left) and human lung implants (right). B) Feature plots showing expression of representative genes enriched in DC subsets within human lung implants. C) Ligand‐receptor signaling intensity between DC subsets (as sender cells) and T cell subsets (as receivers). D) Schematic of the experimental design. Dual‐humanized mice were exposed to HKU1 for 14 days. Human lung implants were then harvested, and antigen‐specific T cells were expanded ex vivo with HKU1 or SARS‐CoV‐2 peptide pools for 16 days. E,F) Representative flow cytometry plots showing virus‐specific (E) CD4^+^ T cells, and (F) CD8^+^ T cells, detected by intracellular cytokine staining (ICS). n = 3 biological replicates.

To evaluate the possibility of CCCoV‐specific antiviral immunity in dual‐humanized mice, we focused on DCs, given their central role as antigen‐presenting cells that initiate and shape T cell responses. Single‐cell transcriptomic analysis revealed the presence of conventional dendritic cells (cDC) subsets (cDC1 and cDC2) and plasmacytoid dendritic cells (pDCs) in human lung implants, with cDC2 being the predominant population, whereas these subsets were largely absent from murine lung tissue (**Figure** **7**A). Notably, human lung‐derived DCs exhibited a mature and immunocompetent phenotype, marked by elevated expression of major histocompatibility complex (MHC) class II molecule HLA‐DQB1 and transcription factors IRF8 and SIRPA (Figure 7B), indicative of robust antigen‐processing and presenting capacity. In addition, innate immune sensors such as toll like receptors (TLR) 4 and TLR 8‐known to detect viral pathogen‐associated molecular patterns including envelope components and single‐stranded RNA, respectively^[^ ^44^ ^]^‐were selectively enriched in the human DC subsets, especially cDC2s (Figure 7B). Ligand‐receptor interaction analysis further demonstrated that cDC2s exhibited the strongest and broadest crosstalk with multiple T cell subsets‐particularly TRMs and memory T cells‐through co‐stimulatory (e.g., TNFSF12‐TNFRSF25), chemotactic (e.g., CXCL16‐CXCR6, CCL19‐CXCR3), and integrin‐mediated adhesion pathways (e.g., ICAM1‐ITGAL/ITGB2, VCAM1‐ITGA4) (Figure 7C). These findings underscore the formation of functional, human‐specific DC‐T cell crosstalk within lung implants, reinforcing the model's probability for mounting viral specific T cell immunity.

Next, we evaluated virus‐specific T cell responses in lung‐immune dual‐humanized mice following HKU1 infection. Human lung implants were harvested 14 days post‐infection for immunological analysis. As in vitro peptide stimulation has proven effective for amplifying low‐frequency T cell responses against allergens and microbial antigens,^[^ ^14^, ^45^, ^46^ ^]^ we applied a 16‐day ex vivo expansion protocol using peptide pools covering the structural proteins (S, N, M, and E) derived from either HKU1 or SARS‐CoV‐2 (Figure 7D). The expanded T cells were then re‐stimulated with overlapping peptide pools of both viruses to assess antigen specificity. Intracellular cytokine staining for interferon‐γ (IFN‐γ) and tumor necrosis factor‐α (TNF‐α) was used to determine whether the antigen‐responsive cells were CD4^+^ or CD8^+^ T cells. In HKU1‐infected lung implants, CD4⁺ T cell responses were predominantly directed against the M and/or E proteins, with IFN‐γ^+^TNF‐α^+^ double‐positive cells reaching approximately 0.38%, compared to less than 0.01% mock control (Figure 7E). CD8^+^ T cell responses were weaker but detectable, mainly targeting the S2 and M and/or E peptide pools (Figure 7F). Notably, we also observed low‐level cross‐reactive CD8 but not CD4 T cell responses to SARS‐CoV‐2 antigens, particularly against the M and/or E proteins, with cytokine production approximately sevenfold above background. These results provide direct experimental evidence that HKU1 infection can elicit T cell responses cross‐reactive with SARS‐CoV‐2. This establishes a physiologically relevant platform for dissecting human T cell immunity to respiratory viruses and offers a valuable tool for pan‐coronavirus vaccine research.

## 3 Discussion

Lung‐humanized mouse models offer a powerful platform to study respiratory pathogens, including viral replication, immune interactions, and human‐specific therapies. However, their reliance on freshly isolated human fetal lung tissue limits scalability and flexibility. Cryopreservation provides a practical alternative for long‐term tissue storage, though concerns remain about its impact on viability and regenerative potential. To address this, we applied an optimized engraftment strategy combining growth factor supplementation (FGF and VEGFA) with standard tissue fragment. Under these optimized conditions, tissues preserved in liquid nitrogen for at least one year exhibited comparable engraftment capacity and growth potential to those stored for three months, supporting the stability and functionality of cryopreserved tissues within this timeframe. This approach significantly enhanced early proliferation, alveolar differentiation, and overall implant maturation, demonstrating that cryopreserved fetal lung tissue can support effective and reproducible engraftment in immunodeficient mice.

While FGF and VEGFA possess relatively short half‐lives in vivo and primarily influence early phase of implant integration, theoretical risks such as excessive proliferation or aberrant differentiation exist. Encouragingly, no histological or functional abnormalities were detected up to 24 weeks post‐transplantation, though longer‐term monitoring will be essential. Interestingly, even without exogenous growth factors, four transplanted fragments could fuse subcutaneously, consistent with previous observations using fresh human fetal lung tissue,^[^ ^19^ ^]^ indicating an inherent integration capacity. Growth factor supplementation further enlarged the fused mass, likely through synergistic mechanisms including enhanced proliferation and survival of lung cells, accelerated establishment of vascular connections with the host to improve perfusion and nutrient supply, and remodeling of the extracellular matrix and microenvironment to reduce barriers between fragments. Notably, FGF and VEGFA, as classical pro‐angiogenic factors,^[^ ^47^, ^48^ ^]^ may act not only on human lung implants but also on murine vasculature, potentially accelerating vascular integration and promoting graft survival. The underlying mechanisms of such cross‐species interactions remain to be explored. While this study focused on FGF and VEGFA, other pathways such as EGF and Wnt signaling,^[^ ^27^ ^]^ which are critical in fetal lung development, may offer additional benefits and warrant further investigation.

Due to species‐specific tropism, conventional laboratory animals such as mice are not naturally susceptible to most CCCoVs. While OC43 can infect mice, it predominantly targets the central nervous system in neonatal mice rather than the respiratory tract.^[^ ^49^, ^50^ ^]^ Other CCCoVs, such as 229E and NL63, require the expression of human entry receptors (e.g., hAPN, hACE2) to establish infection in mice.^[^ ^16^ ^]^ OC43 and HKU1 utilize 9‐O‐acetylated sialic acids as entry receptors,^[^ ^51^ ^]^ limiting the utility of receptor‐transgenic strategies. Although TMPRSS2 has been proposed as a functional co‐receptor for HKU1,^[^ ^52^, ^53^ ^]^ no permissive mouse model has been reported to date. Our lung‐humanized platform therefore offers a unique advantage by enabling side‐by‐side comparison of all four CCCoVs and SARS‐CoV‐2 under consistent physiological conditions. Notably, SARS‐CoV‐2 exhibited a broader target cell range‐from alveolar to airway region‐and induced more severe pathological changes compared to other CCCoVs, which may explain its higher pathogenicity. Our SARS‐CoV‐2 challenge data further demonstrate that growth factor treated cryopreserved human fetal lung tissues respond similarly to fresh tissue,^[^ ^26^ ^]^ validating their utility for infection studies and supporting the robustness of this platform.

To characterize these tropism patterns, we analyzed the expression of reported coronavirus entry receptors and cofactors in our single‐cell dataset. As expected, ACE2 was enriched in AT2 cells, consistent with the colocalization of SARS‐CoV‐2 antigens with AT2. However, a subset of virus‐positive cells lacked ACE2 expression, suggesting ACE2‐independent entry. Alternative receptors such as ASGR1, KREMEN1, and AXL were broadly expressed across AT1, AT2, Club, ciliated, endothelial, and mesenchymal compartments, consistent with our immunofluorescence results and supporting expanded tropism.^[^ ^54^, ^55^ ^]^ Among the four CCCoVs, NL63, although an *alphacoronavirus*, also utilizes ACE2 as its entry receptor, similar to the *betacoronavirus* SARS‐CoV‐2. In our experiments, NL63 predominantly infected airway epithelial cells rather than alveolar epithelium, implying the involvement of additional cofactors. The 229E receptor ANPEP appeared enriched in endothelial cells in our single‐cell dataset, while immunofluorescence staining demonstrated that 229E primarily infected airway epithelial and mesenchymal cells. This discrepancy may reflect differences between transcript and protein expression patterns, warranting further investigation. It is also possible that other host entry factors contribute to the observed 229E tropism in our model. The functional receptor for OC43 has not yet been fully reported; however, HKU1 has been shown to exploit TMPRSS2, similarly to SARS‐CoV‐2.^[^ ^52^, ^53^ ^]^ In our dataset, TMPRSS2 was expressed in AT1, AT2, Club, and ciliated cells, aligning with our observation that HKU1 primarily infected Club and ciliated cells, while SARS‐CoV‐2 exhibited partially overlapping infection patterns in airway epithelium.

Our findings highlight the importance of human stromal and immune cell interactions in establishing a functional lung‐resident immune compartment. Human macrophages were detected exclusively in lung implants but not in murine lungs, suggesting that their differentiation is spatially restricted and driven by human‐specific niche signals. A previous study using humanized MISTRG mice, which express human macrophage colony‐stimulating factor, granulocyte‐macrophage colony‐stimulating factor (GM‐CSF), and IL‐3, shows that CD14^+^ monocytes from hematopoietic stem and progenitor cells can infiltrate lung tissue and differentiate into marophages.^[^ ^56^ ^]^ Although our model does not transgenically express human GM‐CSF, a key factor regulating alveolar macrophage development via PPARγ,^[^ ^57^ ^]^ various human stromal cells in the lung implants, including epithelial and endothelial cells, are capable of secreting GM‐CSF.^[^ ^58^, ^59^ ^]^ Compared with transgenic human cytokine‐expressing models, our lung‐humanized system more faithfully recreates the human pulmonary microenvironment, thereby better supporting the differentiation and function of human lung macrophages. TRM, enriched in mucosal epithelial layers, act as frontline defenders by rapidly responding to infection and orchestrating downstream innate and adaptive immune responses.^[^ ^60^, ^61^ ^]^ In addition to their role in infection, TRMs have also been associated with improved cancer survival,^[^ ^62^, ^63^, ^64^ ^]^ although the mechanisms underlying their anti‐tumor effects remain unclear. In our study, both flow cytometry and single‐cell transcriptomic analyses revealed that TRMs are specifically enriched in human lung implants but absent from murine lung. While previous studies using fresh human fetal lung tissues in BLT mice also reported TRM presence,^[^ ^23^ ^]^ they did not include direct comparison with mouse lungs or investigate regulatory mechanisms. Our single‐cell transcriptomic data identified alveolar fibroblasts and FOLR2⁺ macrophages as the principal cell types interacting with TRMs. FOLR2⁺ macrophages provided a rich repertoire of immunomodulatory signals, while alveolar fibroblasts contributed adhesive support, particularly via integrin‐mediated pathways. Together, these findings establish our dual‐humanized model as a powerful platform for investigating the development, regulation, and function of human tissue‐resident immune cells within the pulmonary niche. Future integration with spatial transcriptomics will help further dissect the niche‐specific regulation and functional roles of human lung macrophages and TRMs in vivo.

Our lung‐immune dual‐humanized mouse model successfully recapitulated human virus‐specific T cell responses and provide direct in vivo evidence that HKU1 infection can elicit T cell responses cross‐reactive with SARS‐CoV‐2. Several factors may contribute to this outcome. First, the use of mTSLP‐transgenic mice likely enhanced lymph node development and supported the generation of antigen‐specific responses.^[^ ^37^ ^]^ Future studies will investigate the immune activity within these lymphoid structures and evaluate their role in initiating pathogen‐specific antiviral immunity. Second, the human lung implants in our model harbored abundant DCs, particularly the cDC2 subset, which expressed elevated levels of MHC class II molecules, TLR4, and TLR8. This enhanced antigen‐sensing and presenting capacity likely contributed to the robust CD4^+^ T cell responses observed against HKU1 M and/or E proteins. We also detected low‐level cross‐reactive CD8 T cell responses to SARS‐CoV‐2 M and/or E proteins, consistent with previous reports that CCCoV infections can elicit T cells with cross‐reactivity to SARS‐CoV‐2 in unexposed individuals.^[^ ^8^, ^9^, ^14^ ^]^ Such cross‐reactivity is shaped not only by viral epitope conservation but also by host genetic background‐particularly HLA alleles that influence antigen presentation. For example, HLA‐B*15:01 has been shown to present conserved peptides shared by HKU1 and SARS‐CoV‐2, facilitating cross‐reactive T cell responses.^[^ ^65^ ^]^ Future studies incorporating donors with diverse HLA genotypes will be essential to further elucidate the impact of HLA variation on cross‐reactive immunity.

Due to the current lack of suitable animal models for HKU1, our study primarily focused on characterizing the virus‐specific T‐cell responses induced by HKU1 infection and their cross‐reactivity with SARS‐CoV‐2. Previous studies have reported that SARS‐CoV‐2 reactive T cells are detectable in approximately 50% of unexposed individuals, and many of these cells can cross‐recognize homologous epitopes derived from CCCoVs,^[^ ^9^, ^14^ ^]^ suggesting the presence of cross‐reactive T cells in the human population. Computational predictions further revealed that approximately 48% of SARS‐CoV‐2 CD8⁺ T‐cell epitopes are “public”, with ≈5.4% shared with at least one CCCoV.^[^ ^66^ ^]^ Another study identified 120 experimentally validated SARS‐CoV‐2 CD8⁺ T cell epitopes derived from structural proteins (E, M, N, S) and RNA‐dependent RNA polymerase (RdRp); subsequent sequence‐homology analysis showed that 15, 6, 14, and 12 of these epitopes exhibited high similarity to OC43, NL63, HKU1, and 229E, respectively.^[^ ^67^ ^]^ The study also predicted several potential cross‐reactive epitopes within the M and E proteins across HKU1, OC43, and 229E,^[^ ^67^ ^]^ which is consistent with our observation that HKU1 infection predominantly induces cross‐reactive CD8⁺ T cell responses against M and/or E. Notably, most shared epitopes identified in this study are derived from the evolutionarily conserved RdRp protein.^[^ ^67^ ^]^ However, our study did not assess potential cross‐reactivity to RdRp, which warrants further investigation. While multiple human studies suggest that SARS‐CoV‐2 cross‐reactive T cells may originate from prior CCCoV infections and that cross‐reactive T cell epitopes indeed exist, the undefined infection histories of donors make it difficult to establish direct causal relationships between specific viral strains and T cell responses. Our lung‐immune system dual‐humanized mouse model provides a unique platform to overcome this limitation and will enable future investigations into T cell cross‐reactivity induced by the other three CCCoVs.

## 4 Conclusion

Our study establishes an optimized approach for engrafting cryopreserved human fetal lung tissue. Human lung implants mature subcutaneously and support productive infection by all four CCCoVs and SARS‐CoV‐2. When combined with immune reconstitution, this model partially restores the human lung immune microenvironment, enabling the investigation of virus‐specific responses following CCCoVs infection. This platform provides a powerful and versatile tool for dissecting human respiratory pathogen biology, immune dynamics, and therapeutic interventions under physiologically relevant conditions.

## 5 Experimental Section

### Ethics Statement

The human tissue samples used in this study were obtained from Nanjing Drum Tower Hospital (Jiangsu, China), the Affiliated Hospital of Nanjing University Medical School. Prior to sample collection, informed consent was obtained from all patients, and the study was approved by the hospital's ethics committee (Ethics Approval No. protocol #2021‐488‐01/02). All CCCoVs (229E, NL63, OC43, HKU1) experiments were performed in a biosafety level 2 laboratory (BSL‐2) at the Guangzhou Medical University (2022188). Experiments related to authentic SARS‐CoV‐2 were conducted in Guangzhou Customs District Technology Center BSL‐3 Laboratory (IQTC202314).

### Animal

The immunodeficient NOD/ShiLtJGpt‐*Prkdc^em26Cd52^Il2rg^em26Cd22^*/Gpt (NCG, Cat. No. T001475) mice and NOD‐*Prkdc* ^em26Cd52^ *Il2rg* ^em26Cd22^ *kit^em1Cin(V831M^Gpt‐Tg(mTSLP)918/*Gpt (NCG‐X‐TSLP, Cat. No. T050142) mice used in this study were obtained from GemPharmatech (Jiangsu, China). The mice were housed in a specific pathogen‐free animal facility with controlled temperature of 22 ± 2 °C, humidity of 55 ± 10%, and a 12 h light/12 h dark cycle. They were provided with standard rodent food and water ad libitum. All procedures involving the generation and use of humanized mice were approved by the Institutional Animal Care and Use Committee (IACUC) of the Model Animal Research Center, Nanjing University Medical School (Approval No. AP# LY‐02).

### Generation of Lung Humanized Mice

Human fetal lung tissues (gestational age 16–22 weeks) were obtained from Nanjing Drum Tower Hospital with ethical approval (Ethics ID: #2021‐488‐01/02). Upon receipt, human fetal lung tissues were transferred into DMEM supplemented with 10% fetal bovine serum (FBS; Gibco) and 1% Penicillin‐Streptomycin (P/S; Sango) on ice. Visible connective tissue and major airway structures were carefully removed, and the remaining parenchyma was dissected into approximately 40–50 mm^3^ fragments. These tissue fragments were cryopreserved in freezing medium consisting of 92.5% fetal bovine serum (FBS; Gibco) and 7.5% dimethyl sulfoxide (DMSO; Sigma) and stored in liquid nitrogen for periods ranging from three months to one year prior to transplantation. Prior to transplantation, cryopreserved tissues were thawed and trimmed into smaller fragments. Either one or four tissue fragments were embedded in 30 µL of Matrigel (Yeason) per flank. For the growth factors‐supplementation group, human basic fibroblast growth factor (bFGF, 1 µg per flank; Sino Biological) and human vascular endothelial growth factor A (VEGFA, 200 ng per flank; Sino Biological) were added to the Matrigel mixture. Lung humanized mice were generated by subcutaneous implantation of the prepared human fetal lung tissue fragments into the dorsal flanks of male and female 4‐ to 6‐week‐old NCG mice, resulting in two independent human lung implants per mouse.

### Generation of Lung‐Immune System Dual‐Humanized Mice

Human fetal livers and lungs (16‐22 weeks of gestational age) were obtained from Nanjing Drum Tower Hospital with ethical approval (Ethics ID: #2021‐488‐01/02). Human CD34^+^ HSCs were isolated from the fetal livers using a CD34^+^ HSCs isolation kit (Miltenyi Biotec, Cat. No. 130‐046‐703) according to the manufacturer's protocol. Male and female neonatal NCG‐X‐TSLP mice (4–7 days old) were used as recipients for intrahepatic injection of 5 × 10^4^ CD34^+^CD38^−^ HSCs per pup, following previously described protocols.^[^ ^68^ ^]^ Dual‐humanized mice were generated by subcutaneous implantation of autologous human fetal lung tissue fragments into NCG‐X‐TSLP mice previously engrafted with human HSCs. Cryopreserved lung tissues were transplanted under optimized conditions: four 2 mm fragments per dorsal flank embedded in Matrigel, supplemented with human bFGF (1 µg per flank) and VEGFA (200 ng per flank). Reconstitution of the human immune system was monitored longitudinally via flow cytometry analysis of peripheral blood.

### Human Lung Implant Volume Measurement

The growth of subcutaneously transplanted human fetal lung tissue was monitored by measuring the size of the subcutaneous bulge. The longest dimension of the implant was defined as the length, and the perpendicular dimension as the width. Both measurements were obtained using a digital caliper. Implant volume was calculated using the ellipsoid formula: Volume = 1/2 × length × width × width.

### Chemicals and Peptides

Paxlovid (Cat. No. 2628280‐40‐8) was purchased from Guangzhou Ruio Biology Science and Technology Co., Ltd, in China. A set of 20‐mer peptides overlapping by 10 amino acids encompassing the HKU1 (GenBank: KF430198.1) and SARS‐CoV‐2 (NC_045512.2) structural proteins [S1, S2, N, and M/E encompassing the N‐ and C‐terminal portions of the spike (S) glycoprotein, the nucleocapsid (N) protein, and the transmembrane (M) and envelope (E) proteins] were synthesized by GL Biochem. Ltd. in China. Specifically, for HKU1, 75 peptides for S1, 61 for S2, 43 for N, 22 for M, and 8 for E were included; for SARS‐CoV‐2, 136 peptides for S1, 117 for S2, 82 for N, 43 for M, and 13 for E were synthesized. These peptides were pooled into S1, S2, M/E, and N libraries and used for T cell stimulation.

### Viruses and Cells

The NL63 strain (NR‐470) was obtained from BEI, and cultured on LLC‐MK2 cells (ATCC, Cat. No.CCL‐7. RRID: CVCL_3009). The 229E strain (ATCC VR‐740) was cultured on MRC‐5 cells (ATCC, Cat. No. CCL‐171. RRID: CVCL_0440). The OC43 strain (ATCC VR‐759) was propagated using suckling mice. The HKU1 strain (KF430198) was cultured using air‐liquid interface cultures of human primary proximal airway organoids. These organoids were established from human fetal lung tissue. The SARS‐CoV‐2 (GenBank: MT123291) was cultured on VeroE6 cells (ATCC, Cat. No. CRL‐1586. RRID: CVCL_0574). All cell lines were obtained from authenticated sources and were confirmed to be free of mycoplasma contamination using the MycAway Plus‐Color One‐Step Mycoplasma Detection Kit (Yeason, Cat. No. 40612ES25) prior to use in infection experiments. Organoid cultures were also regularly monitored for contamination and abnormal morphology, and were found to be free of mycoplasma.

### CCCoV and SARS‐CoV‐2 Infection and Antiviral Treatment

At 8–12 weeks post‐transplantation, lung‐humanized mice were randomly assigned to experimental groups infected via direct intra‐implant injection. Mice were inoculated with one of the following human coronaviruses: 229E (3 × 10^4^ focus‐forming units [FFU], 1.95 × 10^10^ copies), NL63 (1.75 × 10^4^ plaque‐forming units [PFU], 3.09 × 10^10^ copies), OC43 (2 × 10^5^ FFU, 5.62 × 10^8^ copies), HKU1 (4.4 × 10^6^ copies), or SARS‐CoV‐2 (5 × 10^4^ FFU), in a total volume of 50 µL of DMEM. Animals were euthanized and human lung implants were harvested at either 2 or 7 days post‐inoculation for downstream analysis. For antiviral evaluation, mice were infected with HKU1 (4.4 × 10^6^ copies) and treated with Paxlovid or vehicle by oral gavage. For each preparation, 0.09 g of Paxlovid was weighed and dissolved in 1 mL of an aqueous vehicle containing 2% Tween‐80 and 0.5% carboxymethyl cellulose (CMC), yielding a final concentration of 90 mg/mL. Treatment was initiated concurrently with infection and administered every 12 hours for a total of four doses. Paxlovid was given at a dose of 600 mg kg^−1^ per mouse. Mice were sacrificed 48 hours after the first dose, and human lung implants were harvested for subsequent analysis.

### SARS‐CoV‐2 Focus‐Forming Assay (FFA)

Viral titers were determined using a focus‐forming assay on Vero E6 cells. One day prior to infection, cells were plated into 96‐well plates. Serial dilutions of SARS‐CoV‐2 viral stocks or human lung implants homogenates were applied to the confluent cell monolayers and incubated at 37 °C for 1 hour. After incubation, the inoculum was discarded, and each well was overlaid with 125 µL of 1.6% CMC medium pre‐warmed to 37 °C. Plates were subsequently incubated for 24 hours. Infected cells were then fixed with 4% paraformaldehyde (PFA, Servicebio) and permeabilized using 0.2% Triton X‐100. Viral antigen was detected by staining with a nucleocapsid‐specific primary antibody (Sino Biological, Cat. No. 40143‐T62), followed by an HRP‐conjugated goat anti‐rabbit secondary antibody (Jackson ImmunoResearch, Cat. No. 111‐035‐003). Foci were visualized using TrueBlue Peroxidase Substrate (KPL) and quantified with an ELISPOT reader (Cellular Technology Ltd). Viral titers were reported as FFU per milliliter of supernatant or per gram of lung tissue.

### CCCoVs Titer Detection

Total RNA was extracted from tissues using RNA Purification Kit (EZBioscience). qRT‐PCR (Quantitative Reverse Transcription PCR) was performed using the EZProbe One Step qRT‐PCR Probe Kit (EZBioscience) on a QuantStudio 6 Real‐Time PCR System (Applied Biosystems) to determine viral RNA loads. Primer and probes sequences are shown in Table S1 (Supporting Information).

### Flow Cytometry

Mice were first anesthetized and subjected to cardiac perfusion with cold PBS to remove circulating blood. Following perfusion, mouse lung tissue and human lung implants were collected and enzymatically digested before being passed through a cell strainer for flow cytometry. For all flow cytometry experiments, flowcytometric analysis was performed using a Flow Cytometer (Agilent) and an LSRFortessa (BD Biosciences). Before antibody incubation, Ig‐binding sites were blocked. The following antibodies were used for flow cytometry: L/D‐BV510 (eBioscience, Cat. No.65‐0866‐18), human CD45‐APC‐Cy7 (BD Biosciences, Cat. No.557833), mouse CD45‐PE‐Cy5 (eBioscience, Cat. No.15‐0451‐82), human CD19‐PE‐CF594 (BioLegend, Cat. No. 302252), human CD3‐BV650 (BD Biosciences, Cat. No. 563852), human CD8‐BUV785 (BD Biosciences, Cat. No. 563823), human CD4‐BUV395 (BD Biosciences, Cat. No. 564724), human TCR‐Va7.2‐PE (BioLegend, Cat. No. 351705), human TCR‐γδ‐FITC (BD Biosciences, Cat. No. 559878), mouse CD45‐BV510 (Miltenyi Biotec, Cat. No. 130‐110‐665), human CD3‐APC‐Cy7 (BD Biosciences, Cat. No. 557832), human‐CD56‐BV650 (BD Biosciences, Cat. No. 564057), human CD45RO‐PE‐Cy7 (BioLegend, Cat. No. 304230), human CD69‐APC (BioLegend, Cat. No. 310910), human CD103‐PE (BioLegend, Cat. No. 350206), human CD4‐FITC (BioLegend, Cat. No. 317408), human CD10‐PE‐Cy7 (BioLegend, Cat. No. 312214), human CD24‐FITC (BioLegend, Cat. No. 311104), human CD38‐PE (BioLegend, Cat. No. 303506), human IgD‐BV421 (eBioscience, Cat. No.62‐9868‐42), human CD27‐BV650 (BD Biosciences, Cat. No. 563228).

### Intracellular Cytokine Staining (ICS)

Cells were stimulated with PMA, ionomycin, and brefeldin A (BFA) (BioGems) at 37 °C for 4 hours. After stimulation, cells were fixed and permeabilized using the Transcription Factor Staining Buffer Set (eBioscience, Cat. No. 00‐5523‐00), followed by staining with the following antibodies: L/D‐BV510 (eBioscience, Cat. No.65‐0866‐18), mouse CD45‐BV510 (Miltenyi Biotec, Cat. No. 130‐110‐665), human CD45‐PE‐Cy5 (eBioscience, Cat. No. 15‐0459‐42), human FOXP3‐APC (BioLegend, Cat. No. 320014), human CD8‐PE‐CF594 (BD Biosciences, Cat. No.562282), human TNF‐α‐BV650 (eBioscience, Cat. No. 416‐7349‐42), IFN‐γ‐PerCP/Cyanine5.5 (BioLegend, Cat. No. 502526), human IL‐13‐BV421 (BioLegend, Cat. No. 501916), human IL‐17A‐BV785 (BioLegend, Cat. No. 512338), human IL‐2‐FITC (eBioscience, Cat. No. 11‐7029‐42), human IL‐4‐PE‐Cy7 (eBioscience, Cat. No. 25‐7049‐82)

### Histology and Immunohistochemical Analysis

Human lung implants were fixed in 4% PFA, embedded in paraffin, and sectioned at 5 µm thickness. Hematoxylin and Eosin (H&E) staining (Sangon, Cat. No. A600701 and A600190) was performed according to the manufacturer's instructions. For immunofluorescence (IF) analysis, deparaffinized sections were subjected to heat‐mediated antigen retrieval using Improved Citrate Antigen Retrieval Solution (Beyotime, Cat. No. P0083) at 95 °C for 30 minutes, followed by cooling to room temperature for 1 hour. Nonspecific binding was blocked with 2% bovine serum albumin (BSA; Beyotime, Cat. No. ST023) for 1 hour. Sections were then incubated with primary antibodies overnight at 4 °C, followed by incubation with fluorescent secondary antibodies for 1 hour at room temperature. For immunohistochemistry staining (IHC), endogenous peroxidase activity was quenched using 3% hydrogen peroxide, followed by blocking of nonspecific binding. Sections were incubated with primary antibodies overnight, then with horseradish peroxidase (HRP)‐conjugated secondary antibodies for 1 hour at room temperature. Signal was visualized using diaminobenzidine (Maixin Biotechnology). For multiplex immunohistochemistry, antigen retrieval, protein blocking, and peroxidase blocking were performed as described above. After incubation with primary antibodies and HRP‐conjugated secondary antibodies, slides were treated with tyramine signal amplification‐conjugated fluorophores, including PPD520 and PPD570 (Panovue, Cat. No. 10217100020), for 3–5 minutes at room temperature. Following each staining cycle, antigen retrieval was repeated to strip bound antibodies while preserving deposited fluorophores, allowing sequential staining of additional targets. Fluorescence imaging was conducted using the Leica TCS SP5 confocal microscope and Olympus SLIDEVIEW VS200 slide scanner. Data statistics were performed using Image‐Pro Plus version 6.0. The primary antibodies used were as follows: SFTPC (Abcam, Cat. No. ab114293), Ki‐67 (Abcam, Cat. No. ab16667), PDPN (MBL, Cat. No. D320‐3), CK19 (Novusbio, Cat. No. NBP1‐78278), Vimentin (Abcam, Cat. No. ab8069), CD34 (Abcam, Cat. No. ab8536), FOXJ1 (Abcam, Cat. No. ab235445), CC10 (Santa Cruz, Cat. No. sc‐365992), CK5 (Abcam, Cat. No. ab52635), CD31 (eBioscience, Cat. No. 12‐0319‐41, 11‐0311‐82), MUC5B (Abcam, Cat. No. ab315330), 229E (Sino Biological, Cat. No. 40640‐T62), NL63 (Sino Biological, Cat. No. 40641‐T62), OC43 (Sino Biological, Cat. No. 40643‐T62), HKU1 (Sino Biological, Cat. No. 40642‐T62), CD20 (Abclonal, Cat. No. A4893), CD68 (Abcam, Cat. No. ab955), CD4 (Abcam, Cat. No. ab133616), CD8 (Abcam, Cat. No. ab60076).

### RNA Extraction and Qpcr

Total RNA was isolated from cells and tissues using TRIzol (Invitrogen, Cat. No. 15596018CN) according to the manufacturer's protocol. Complementary DNA (cDNA) was synthesized using a Reverse Transcription Reagent Kit (Yeason, Cat. No. 11141ES10). qPCR was performed using SYBR Green Master Mix (Yeason, Cat. No. 11201ES03) with a Roche LightCycler 96 (Roche Applied Science). Gene expression levels were normalized to the housekeeping gene *GAPDH*. The average Ct value of *GAPDH* was subtracted from the average Ct value of the target gene. The normalized values, presented as 2^−ΔΔCt^, were used to analyze the relative expression levels of the target genes. Primer sequences are shown in Table S2 (Supporting Information).

### Single‐Cell RNA Sequencing

Following tissue harvest from one lung‐immune dual‐humanized mouse (n = 1), both human lung implants and murine lung tissues were mechanically and enzymatically dissociated into single‐cell suspensions using a tissue dissociation kit (RWD, Cat. No. DHGT‐5004) according to the manufacturer's instructions. The resulting cell suspensions were filtered through a 40 µm cell strainer, centrifuged, and resuspended in appropriate buffer. The DNBelab C Series Single‐Cell Library Prep Set (MGI, 1000021082) was utilized for scRNA‐seq library preparation,^[^ ^69^ ^]^ involving droplet encapsulation, bead collection, reverse transcription, cDNA amplification, and purification. cDNA was sheared into 250–400 bp fragments, and indexed libraries were made following the protocol. Quantification used the Qubit ssDNA Assay Kit (Thermo Fisher Scientific, Q10212) and Agilent Bioanalyzer. Libraries were sequenced on a DNBSEQ‐T1 sequencer at the Biopharmaceutical Public Service Platform (Nanjing Jiangbei New Area, China) with the following sequencing strategy: paired‐end sequencing, including a 30 bp read for barcodes and unique molecular identifier (UMI), a 100 bp read for cDNA.

### scRNA‐Seq Data Processing

The sequencing data were processed using informal testing pipeline (https://github.com/MGI‐tech‐bioinformatics/DNBelab_C_Series_HT_scRNA‐analysis‐software/issues/165). Briefly, all samples underwent read processing, alignment with the GRCh38 and GRCm38 transcriptome, UMI counting, and droplet partition. Main downstream analyses of the human lung tissue cell gene count matrix and the mouse lung tissue cell gene count matrix were performed using Seurat (v5.2.1) package in R (v4.4.1).^[^ ^70^ ^]^ Genes detected in fewer than 3 cells were filtered out prior to further analyses. The initial filtration of human cells used the “percent.mt < 10” and “nFeature_RNA > 200” thresholds. For scRNA‐seq data of mice cells, cells with fewer than 200 genes detected or with percent.mt higher than 5% were filtered. After cell filtration, UMI data was normalized using the default method “LogNormalize” to remove the influence of technical effects in the underlying molecular counts. The top 2000 most variable genes were found by using the function FindVariableFeatures and then used for principal component analysis (PCA). Function ScaleData was used to standardize and centralize the data. Next, PCA was performed on the scaled data. The ElbowPlot function was used to sort the principal components according to the percentage of variance explained by each one to determine how many top principal components were chosen to cluster the cells. The first 20 principal components and resolution 1.0 were used in the FindNeighbors and FindClusters functions. And the uniform manifold approximation and projection (UMAP) dimensional reduction technique was applied to visualize cell clusters. DoubletFinder (v2.0.3) R package was used to detect doublets in each sample.^[^ ^71^ ^]^ Since the samples of human lung tissue and mice lung tissue come from different donors, this may introduce technical and biological batch effects in downstream analysis. Therefore, the study used the IntegrateLayers function of Seurat to deal with the batch effect through the built‐in Harmony integration method (method = HarmonyIntegration). Human and mouse cells underwent quality control filtering as described previously and merged into one object, retaining only homologous genes and performing the same processing steps.

### Cell Type Annotation

For human cells, clusters were annotated according to the distinct expression patterns of the following reported cell markers:^[^ ^72^ ^]^ AT1 (*AQP5*, *PDPN*, *AGER*), AT2 (*SFTPA1*, *SFTPB*, *SFTPC*, *ABCA3*), Basal cell (*KRT5*, *TP63*), Club cell (*SCGB1A1*), Ciliated cell (*DYNLRB2*, *SNTN*, *C9orf24*, *CDHR3*, *DYDC2*), Smooth muscle cell (*DES*, *CNN1*, *TAGLN*, *ACTA2*), Pericyte (*MCAM*, *PDGFRB*, *NG2*, *RGS5*), Alveolar fibroblast (*DCN*, *COL5A1*, *LUM*, *COL6A3*, *COL6A1*), Chondrocyte (*COL2A1*, *COL11A2*, *ACAN*), Endothelial (*RNASE1*, *PCDH17*, *ADGRL4*, *PECAM1*, *VWF*), Immune cells (*PTPRC*), and Proliferating cell (*MKI67*, *PCNA*, *TOP2A*). Immune cells were identified for downstream analysis and performed the same processing steps and further clustering. Subsequently, all immune cell clusters were annotated in accordance with the markers documented in previous reports:^[^ ^72^ ^]^ T cell (*CD3D*, *CD3E*, *CD3G*), B cell (*CD79A*, *CD19*), NK cell (*KLRD1*, *NCAM1*, *EOMES*), Monocyte (*CD14*, *LYZ*, *VCAN*), Macrophage (*C1QA*, *ITGAM*, *VCAM1*, *MRC1*, *ADGRE1*), cDC1 (*CLEC9A*), cDC2 (*CD1C, PPA1*, *FCER1A*), pDC (*IRF8*, *IRF7*, *LILRA4*, *TCF4*), Mast cell (*KIT*, *TPSAB1*, *HPGD*, *TPSB2*, *MS4A2*, *GATA2*), and Neutrophil (*S100A8*, *S100A9*, *CSF3R*, *G0S2*).

For mice cells, clusters were annotated according to the distinct expression patterns of the following reported cell markers:^[^ ^72^ ^]^ Epithelial (*Epcam*), Fibroblast (*Col1a1*), Endothelial (*Pecam1*), Immune cells (*Ptprc*), Erythroid cell (*Hbb‐bt*, *Hbb‐bs*, *Hba‐a1*), Platelet (*Pf4*, *Gp9*, *Itga2b*, *Ppbp*), Neuron (*Rbfox1*, *Nrxn3*, *Dab1*, *Pcdh15*), Neuroendocrine (*Sox10*, *Foxd3*, *Ncam1*, *S100b*), and Proliferating cell (*Mki67*, *Top2a*, *Pcna*). Stromal cells were identified for downstream analysis and performed the same processing steps and further clustering. Subsequently, all stromal cell clusters were annotated in accordance with the markers documented in previous reports:^[^ ^72^ ^]^ AT1 (*Ager*, *Clib5*, *Pdpn*), AT2 (*Sftpc*, *Muc1*, *Etv5*), Club cell (*Scgb3a2*, *Cyp2f2*), Ciliated cell (*Foxj1*, *Ccdc78*, *Fam183b*), Smooth muscle cell (*Acta2*, *Cnn1*, *Tagln*), Pericyte (*Trpc6*, *Higd1b*, *Vtn*), Myofibroblast (*Wif1*, *Fgf18*, *Aspn*), Alveolar fibroblast (*Fgfr4*, *Slc7a10*, *Slc38a5*), Capillary (*Pvalp*, *Pgihbp1*, *Cd93*), Capillary aerocyte (*Ednrb*, *Car4*, *Prx*), Vein (*Slc6a2*, *Amigo2*, *Vegfc*), Artery (*Gja5*, *Bmx*), and Lymphatic (*Prox1*, *Mmrn1*).

### Cell‐Cell Communication Analysis

In order to investigate cell‐cell interactions between each cell cluster, the ligand‐receptor pairs were analyzed using CellphoneDB (v5.0.0),^[^ ^73^ ^]^ which provides a new database (cellphonedb‐data v5.0) with more manually curated interactions, making up to a total of ≈3000 interactions classified by signaling pathways. Based on the metadata and normalized cell matrix implemented by Seurat, the significant mean and cell communication significance were calculated by method 2 cpdb_statistical_analysis_method. The study calculated the sum of the mean values for significant ligand‐receptor interactions (*p* < 0.05) between stromal cells, immune cells, and TRM, respectively, and displayed it by R package ggplot2 (v3.3.6). The circos plot using R package circlize (v0.4.15) showed significant ligand–receptor interactions between specific cell‐cell interaction pairs.^[^ ^74^ ^]^ The sector color is determined by the classification color, the sector width and color transparency are determined by the mean values of interactions, and the sector direction is directed by the ligand to the receptor. In addition, the R package ggplot2 was used to plot the dot plot of the ligand‐receptor interactions between DC‐T cell interaction pairs, in which the significant ligand‐receptor interactions were circled with black borders (*p* < 0.05).

### Data Availability

The raw sequence data reported in this paper have been deposited in the Genome Sequence Archive (Genomics, Proteomics & Bioinformatics 2025) in National Genomics Data Center (Nucleic Acids Res 2025), China National Center for Bioinformation / Beijing Institute of Genomics, Chinese Academy of Sciences (GSA‐Human: HRA014884) that are publicly accessible at https://ngdc.cncb.ac.cn/gsa‐human.

### Generation of Human Antigen‐Presenting Cells (APCs)

Autologous human antigen‐presenting cells were generated from spleens of humanized mice. Human B cells were enriched from spleen‐derived single‐cell suspensions using anti‐human CD19 MicroBeads (Miltenyi Biotec), according to the manufacturer's instructions. The positively selected CD19⁺ B cells were transformed into B‐lymphoblastoid cell lines (BLCLs) via Epstein‐Barr virus (EBV) infection. Specifically, CD19^+^ cells were incubated overnight with EBV‐containing supernatant in the presence of 2.5 mg mL^−1^ CpG oligodeoxynucleotides (Invitrogen) to enhance transformation efficiency. Cells were then cultured at 37 °C in RPMI 1640 medium supplemented with 10% fetal bovine serum (FBS; Gibco) and expanded over several weeks. Successfully transformed BLCLs were maintained and used as autologous APCs for downstream functional assays.

### Detection of Virus‐Specific and Cross‐Reactive T Cells

Lung‐immune dual‐humanized mice were infected via intra‐implant injection with HKU1 (4.4 × 10^6^ copy) or vehicle. Fourteen days post‐infection, human lung implants were harvested, and single‐cell suspensions were prepared. Autologous BLCLs, generated as previously described, were irradiated at 80 Gy. After washing, BLCLs were pulsed with 2 µm peptide pools derived from either HKU1 or SARS‐CoV‐2 and incubated on a shaker at 37 °C for 1–2 hours to allow antigen loading. Peptide‐pulsed BLCLs were then co‐cultured with lung‐derived lymphocytes (1:2) in the presence of human IL‐2 (20 IU/mL). Cultures were maintained for 16 days, with periodic medium replacement to maintain IL‐2 levels and cell viability. Following the expansion period, T cells were re‐stimulated with the corresponding peptide pools and assessed by ICS. Cells were fixed and permeabilized using BD Cytofix/Cytoperm solution (BD, Cat. No. 554714), then stained with antibodies against IFN‐γ and TNF‐α for flow cytometric analysis.

### Data Analysis and Statistics

All experimental data were analyzed using GraphPad Prism 10 software. Data are presented as mean ± standard deviation (SD), with the number of samples (n) indicated in the text or figure legends. Group comparisons were conducted using unpaired two‐tailed Student's *t*‐test and one‐way ANOVA followed by Tukey's multiple comparison test, as appropriate and described in the respective legends. A significance level of *p* < 0.05 was considered statistically significant.

## Conflict of Interest

Y.L. is the inventor of patents covering the development of humanized mouse models and therapeutic targeting of infection, cancer, and autoimmune disorders. Among these, one patent application on a lung‐humanized mouse model is relevant to this study.

## Author Contributions

C.C., J.S., C.L., and Q.Y. contributed equally to this work. C.C. and Y.L. conceived and designed the study. Y.L., J.Z., and D.S. supervised the project and revised the manuscript. C.C. performed the experiments, analyzed the data, and wrote the manuscript with assistance from Y.Q. and D.R. J.S. carried out all mouse and in vitro experiments related to viral infections in BSL‐2 and BSL‐3 laboratories in China, with support from Y.H. and B.W. L.C. was responsible for the single‐cell RNA sequencing data analysis. Clinical samples were provided by S.D.

## Supporting information



Supporting Information

## Data Availability

The data that support the findings of this study are available from the corresponding author upon reasonable request.
