# Supplementary material for: A Lung‐Immune Dual‐Humanized Mouse Using Cryopreserved Tissue Enables Infection and Immune Profiling of Human Common Cold Coronaviruses
Source: Adv Sci (Weinh). 2025 Dec 7;13(16):e12097. doi: 10.1002/advs.202512097 (PMC13042428; doi:10.1002/advs.202512097)
Supplement: Supplementary file 1 — Supporting Information [file ADVS-13-e12097-s001.docx]

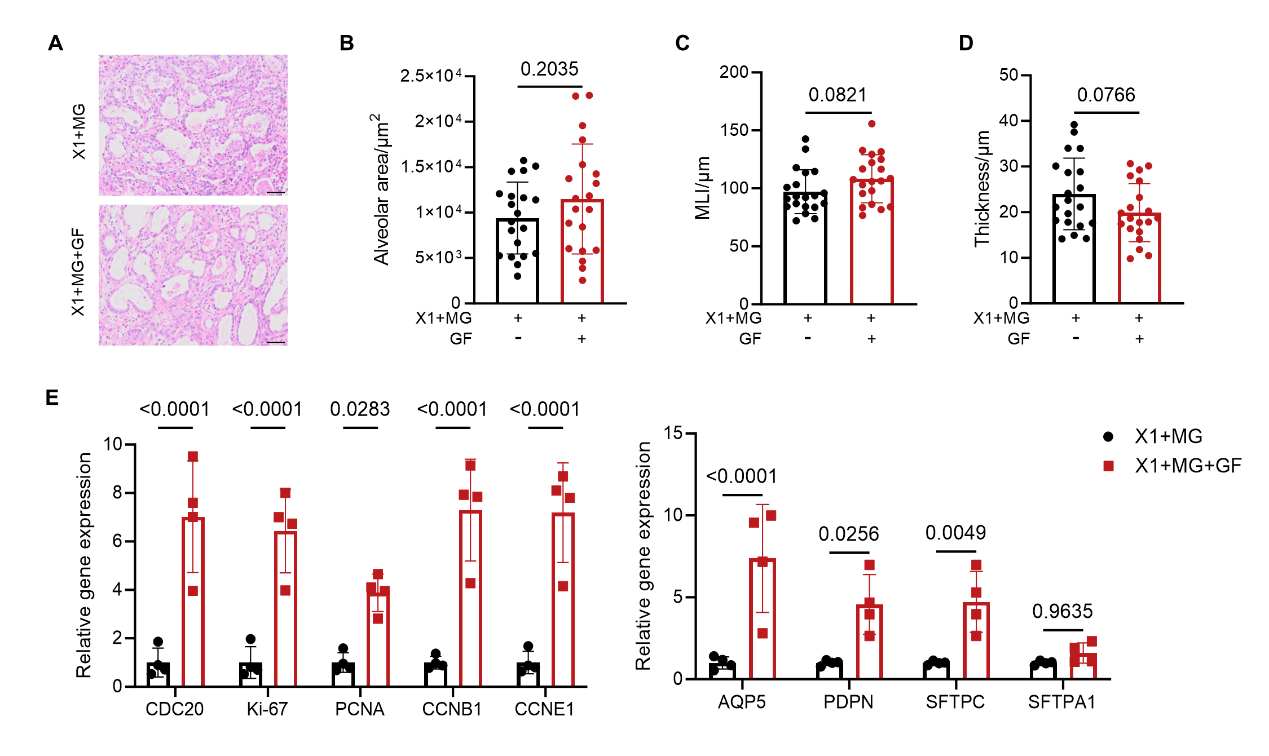
**Extended Data Figure 1.** Growth factors promote proliferation and differentiation of cryopreserved human lung implants. A) H&E staining of human lung implants with or without growth factor treatment one week post-transplantation. Scale bars: 50 µm. B-D) Quantification of B) alveolar area, C) mean linear intercept (MLI) and D) alveolar wall thickness. n = 4 biological replicates, 20 technical replicates. E) qPCR analysis of relative mRNA expression levels for proliferation-related genes (*CDC20*, *Ki-67*, *PCNA*, *CCNB1*, and *CCNE1*) and alveolar differentiation-related genes (*AQP5*, *PDPN*, *SFTPC*, and *SFTPA1*). n = 4 biological replicates. Data presented as means ± SD. Statistical analyses were performed using a nonpaired two-tailed Student’s t-test.


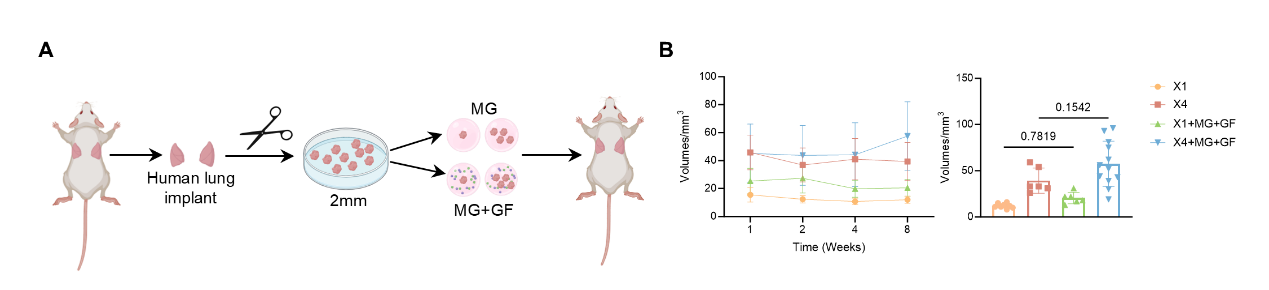
**Extended Data Figure 2.** Serial transplantation of human lung implant. A) Schematic of the serial transplantation procedure. Human lung tissue harvested from established lung-humanized mice was transplanted into recipient NCG mice, with one (×1) or four (×4) tissue fragment per side. Tissues were either untreated (MG) or supplemented with growth factors (MG + GF). B) Dynamic and endpoint volumes of human lung implants 8 weeks after serial transplantation. Group sizes: ×1, n = 8; ×4, n = 6; ×1 + MG + GF, n = 6; ×4 + MG + GF, n = 12. Data are presented as mean ± SD. Statistical significance was determined by one-way ANOVA with Tukey’s multiple comparisons test.


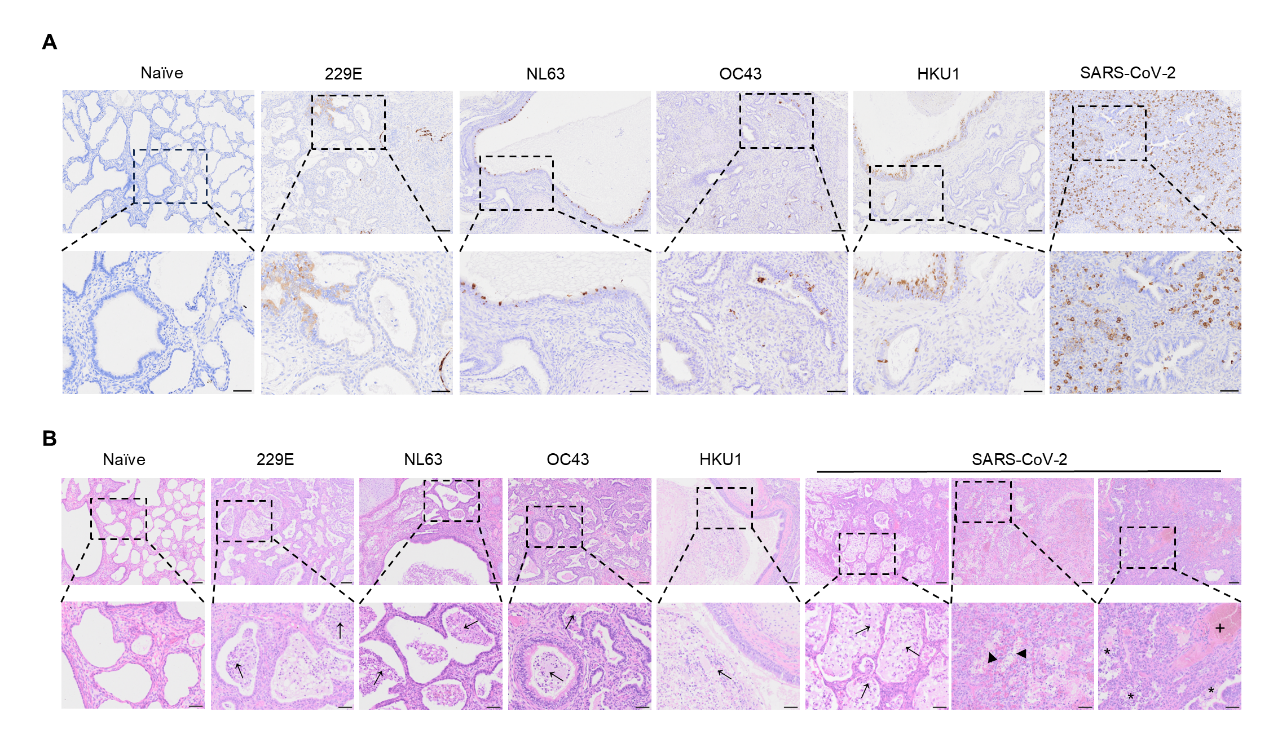
**Extended Data Figure 3.** SARS-CoV-2 exhibits distinct cellular tropism and more severe histopathological changes compared to CCCoVs. A) Immunohistochemistry of viral nucleocapsid proteins in naïve and human lung implants infected with 229E, NL63, OC43, HKU1, and SARS-CoV-2. Scale bars: 100 µm (top) and 50 µm (down). B) H&E staining of naïve and infected human lung implants showing histopathological alterations induced by 229E, NL63, OC43, HKU1, and SARS-CoV-2. Immune cells accumulation and denuded epithelial cells (black arrows) within airway lumen and airspaces, alveolar hemorrhage (triangle), extensive fibrin thrombi occluding blood vessels (plus sign) and the accumulation of necrotic debris within airspaces (asterisks). Scale bars: 100 µm (top) and 50 µm (down).


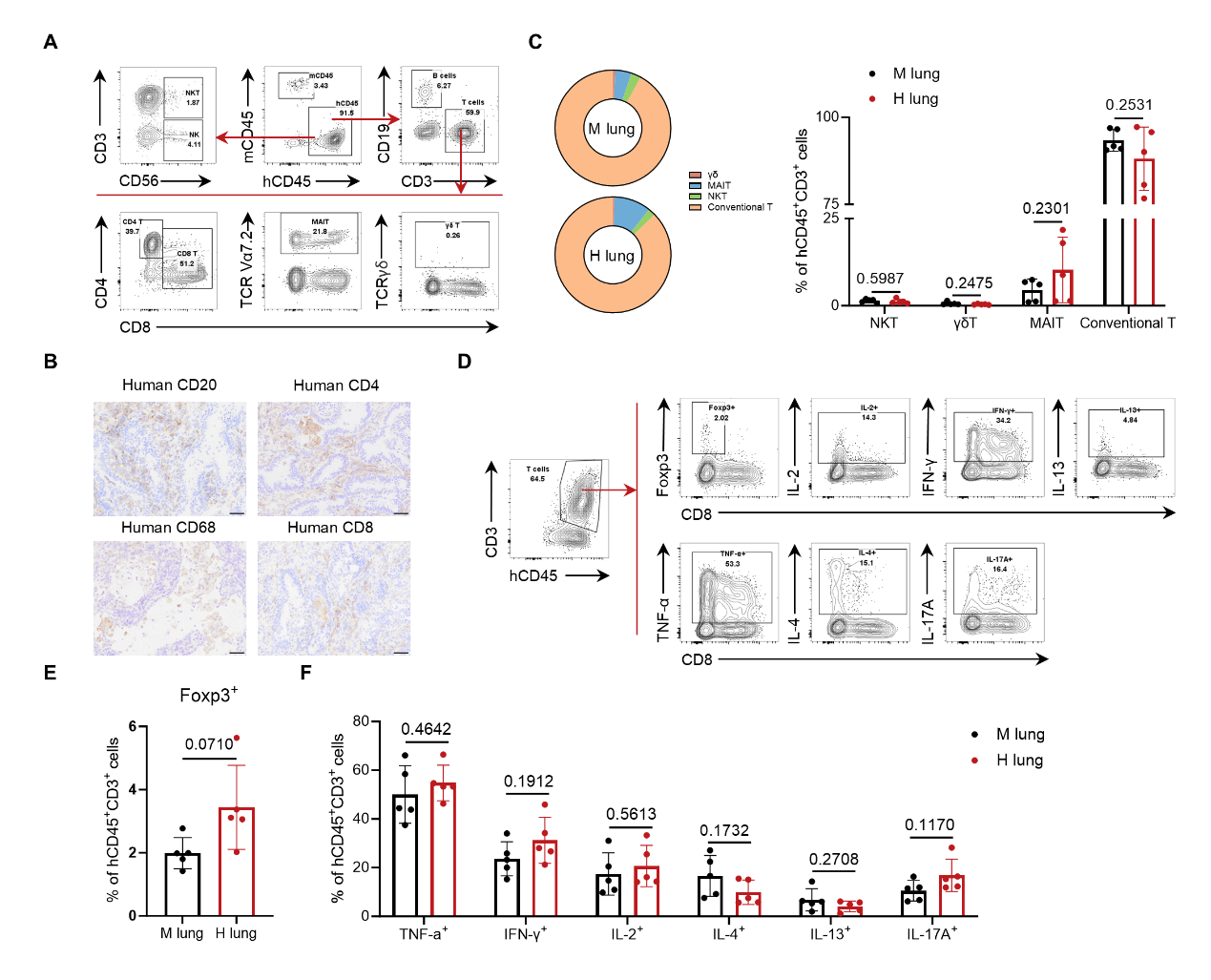
**Extended Data Figure 4.** Characterization of human immune cells and T cell subtypes in lung-immune system dual-humanized mice. A) Gating strategies for identifying human immune cells and T cell subtypes. B) Representative immunohistochemistry staining of human immune cells, including B cells (hCD20^+^), macrophages (hCD68^+^), CD4^+^T cells (hCD4^+^) and CD8^+^T cells (hCD8^+^) in human lung implants. Scale bars: 50 µm. C) The distribution and percentage of human T cell subsets, including conventional T cells, γδ T cells (TCR γδ^+^), mucosal-associated invariant T cells (MAIT, TCR Vα7.2-Jα33^+^) and natural killer T (NKT) cells (hCD56^+^). D) Gating strategies, and E-F) percentage of human regulatory T cells (Tregs) and intracellular cytokine-producing T cells subsets following PMA/Ionomycin stimulation in in both mouse lung and human lung implant. Data presented as means ± SD. Statistical analyses were performed using unpaired two-tailed Student’s t test. n = 5 biological replicates.


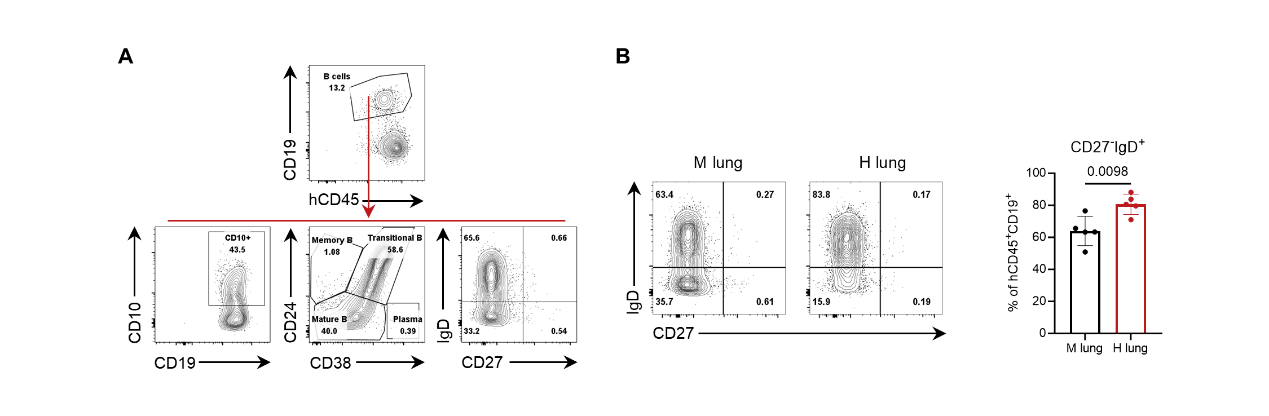
**Extended Data Figure 5.** Enhanced B cell maturation observed in human lung implants. A) Gating strategies for identifying human B cell subtypes. B) Representative flow cytometry analysis and the quantification of human mature B cells (hCD27^-^hIgD^+^) in both mouse lung and human lung implant. n = 5 biological replicates. Data presented as means ± SD. Statistical analyses were performed using unpaired two-tailed Student’s t test.


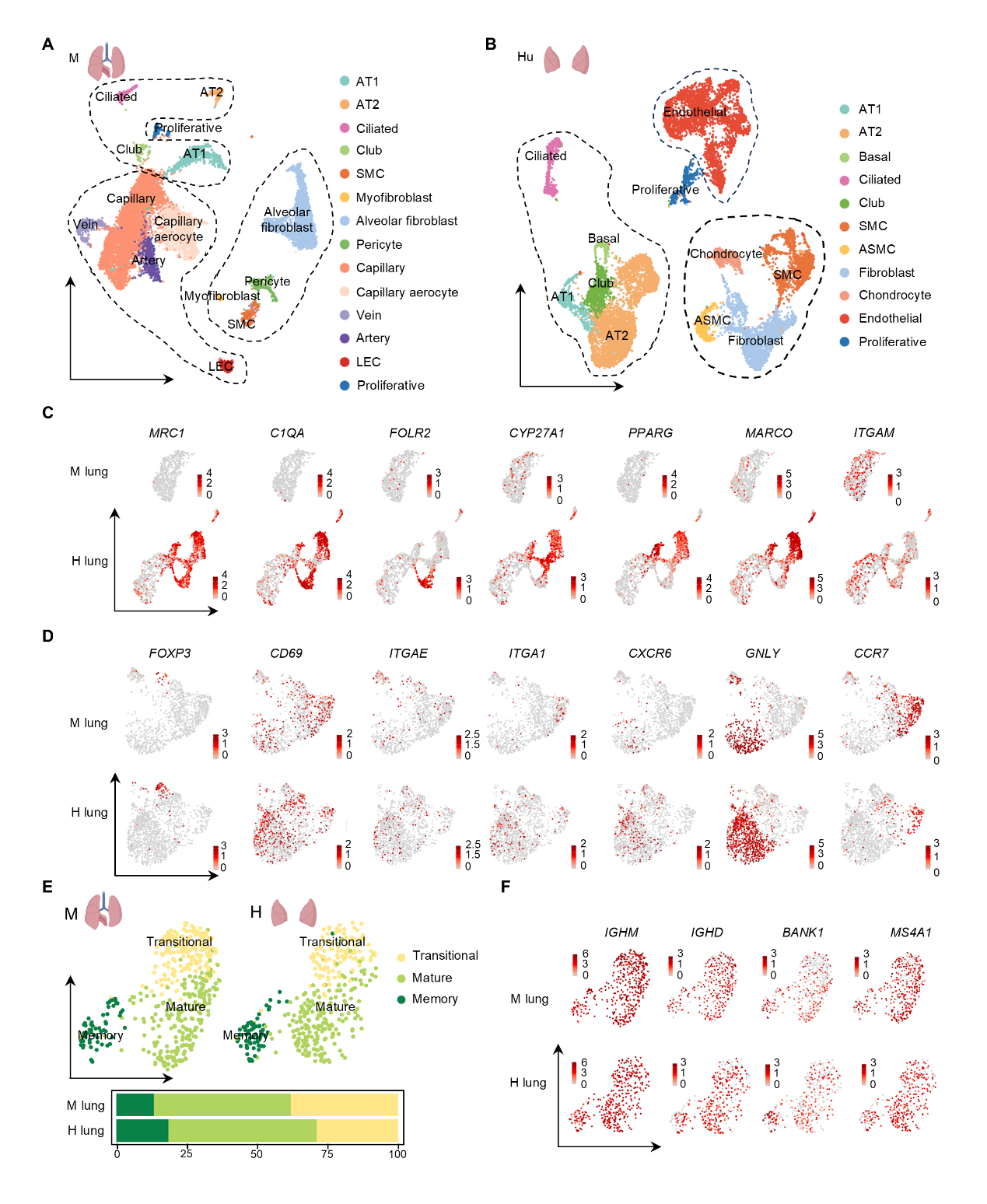
**Extended Data Figure 6.** Identification and transcriptional profiling of lung cell subsets in lung-immune system dual-humanized mice. A-B) Identification of lung stromal cell subsets in A) mouse lungs and B) human lung implants from lung-immune system dual-humanized mice (SMC, smooth muscle cells; LEC, lymphatic endothelial cells; ASMC, arterial smooth muscle cells). C-D) UMAP plots showing the scaled expression of representative marker genes in human C) myeloid cells and D) T cells. E) UMAP plots and subclustering analyses of human B cells in mouse lungs and lung implants. Frequencies of cell subsets are shown below the respective UMAP plots. F) UMAP plots showing the expression of representative marker genes in human B cell populations.

Supplemental Table S1. Primer pairs and probes used for detection of CCCoVs titers

| Target | Primer/Probe | Sequence |
| --- | --- | --- |
| 229E | Forward | CGCAAGAATTCAGAACCAGAG |
| 229E | Reverse | GGGAGTCAGGTTCTTCAACAA |
| 229E | Probe | FAM-CCACACTTCAATCAAAAGCTCCCAAATG-BHQ1 |
| NL63 | Forward | AGGACCTTAAATTCAGACAACGTTCT |
| NL63 | Reverse | GATTACGTTTGCGATTACCAAGACT |
| NL63 | Probe | FAM-TAACAGTTTTAGCACCTTCCTTAGCAACCCAAACA-BHQ1 |
| OC43 | Forward | CGATGAGGCTATTCCGACTAGGT |
| OC43 | Reverse | CCTTCCTGAGCCTTCAATATAGTAACC |
| OC43 | Probe | FAM-TCCGCCTGGCACGGTACTCCCT-BHQ1 |
| HKU1 | Forward | CTTGAGCACATTCATTCGCAAG |
| HKU1 | Reverse | GGTTGGGATTATCCTAAATGTGA |

Supplemental Table S2. Primer pairs used for qPCR

| Target | Forward primer | Reverse primer |
| --- | --- | --- |
| *PDPN* | GTGCCGAAGATGATGTGGTGAC | GGACTGTGCTTTCTGAAGTTGGC |
| *AQP5* | GCTCACTGGGTTTTCTGGGTA | TCCATGGTCTTCTTCCGCTC |
| *SFTPC* | ATGGATGTGGGCAGCAAAGA | CAGCAGGGAATGCCAAATCG |
| *SFTPA1* | CACCTGGAGAAATGCCATGTCC | AAGTCGTGGAGTGTGGCTTGGA |
| *Ki-67* | AGAGGTGTGCAGAAAATCCAA | TGTCCCTATGACTTCTGGTTCTTA |
| *PCNA* | TGGAGAACTTGGAAATGGAAAC | GAACTGGTTCATTCATCTCTATGG |
| *CCNB1* | TTTCGCCTGAGCCTATTTTG | GCACATCCAGATGTTTCCATT |
| *CCNE1* | GGCCAAAATCGACAGGAC | GGGTCTGCACAGACTGCAT |
| *CDC20* | CTGTCTGAGTGCCGTGGAT | TCCTTGTAATGGGGAGACCA |
| *GAPDH* | TCTTCTTTTGCGTCGCCAGCCG | CCCTGCAAATGAGCCCCAGCC |
